# Timed material self-assembly controlled by circadian clock proteins

**Published:** 2023-03-01

**Authors:** Gregor Leech, Lauren Melcher, Michelle Chiu, Maya Nugent, Lily Burton, Janet Kang, Soo Ji Kim, Sourav Roy, Laila Farhadi, Jennifer L. Ross, Moumita Das, Michael J. Rust, Rae M. Robertson-Anderson

**Affiliations:** 1 Department of Physics and Biophysics, University of San Diego, San Diego, California 92110, United States; 2 School of Mathematical Sciences, Rochester Institute of Technology, Rochester, New York 14623, United States; 3 Graduate Program in Biophysical Sciences, University of Chicago, Chicago, Illinois 60637, United States; 4 Department of Biochemistry and Molecular Biophysics, University of Chicago, Chicago, Illinois 60637, United States; 5 Department of Molecular Genetics and Cell Biology and Department of Physics, University of Chicago, Chicago, Illinois 60637, United States; 6 Department of Physics, Syracuse University, Syracuse, New York 13244, United States; 7 School of Physics and Astronomy, Rochester Institute of Technology, Rochester, New York 14623, United States

## Abstract

Biological systems present a powerful, yet largely untapped, opportunity to impart autonomous regulation to materials. Because these systems can function robustly to regulate when and where chemical reactions occur, they have the ability to bring complex, life-like behavior to synthetic materials. Here, we achieve this design feat by using functionalized circadian clock proteins, KaiB and KaiC, to engineer time-dependent crosslinking of colloids. The resulting material self-assembles with programmable kinetics, producing macroscopic changes in material properties, via molecular assembly of KaiB-KaiC complexes. We show that colloid crosslinking depends strictly on the phosphorylation state of KaiC, with kinetics that are synced with KaiB-KaiC complexing. Our microscopic image analyses and computational models indicate that self-assembly of colloidal super-structures requires multiple Kai complexes per colloid connection, which then stabilizes the material against dissolution. This work introduces the concept of harnessing biological timers to control synthetic materials; and, more generally, opens the door to using protein-based reaction networks to endow synthetic systems with life-like functional properties.

The current state-of-the-art in next-generation materials design is to create structures that can achieve desired functions in response to external perturbations, such as self-repair in response to damage. Looking beyond this stimulus-response framework, we envision autonomously functional materials that can not only respond directly to their environment, but also have the capability to store a memory of past events and dynamically change their own properties. Such materials could be used to create dynamic sequestration devices that filter toxins on a programmable schedule, or medical implants that self-assemble and restructure to protect and suture wounds and dissolve once fully healed.

An attractive strategy to equip materials with robust autonomous function is the use of distributed information processing throughout the material, rather than a central controller. This concept is similar to the myriad networks of interacting biomolecules in living cells, which provide finely-tuned spatiotemporal regulation of cellular functionality. In many cases, a small number of interacting network components can be isolated from the cell and retain modular function to achieve tasks such as defining structures with a specific size^1^, generating spatial patterns^2^, or keeping time^3^. On a larger scale, the collective action of these biomolecules provides a way for energy flux to impart non-equilibrium properties into structures to create active matter. The last two decades have seen tremendous progress on identifying and understanding the emergent properties of active matter from active colloids, to molecular motor-driven active biomaterials, to soft robotics and living concrete^4–12^. However, engineering autonomous materials with robust, kinetically-controlled activity, inherent to living systems, remains a grand challenge in materials science^13–15^.

Here, we develop the proof-of-concept of an autonomous material with properties that are temporally programmed by biological signaling molecules using protein components derived from the cyanobacterial circadian clock (Fig. 1). In their natural context, the KaiA, KaiB, and KaiC proteins generate a self-sustaining ~24-hour rhythm that is used to synchronize physiology with the external light-dark cycle. Remarkably, these proteins can be removed from their cellular context and still continue to generate oscillations in a reconstituted in vitro system^3,16–18^.

These oscillations can be detected as an ordered pattern of multisite phosphorylation on KaiC, which acts as a signaling hub that binds and releases protein partners throughout the cycle^19–21^. In brief, KaiC consists of two tandem ATPase domains, CI and CII, arranged into a hexameric ring (one blue circle in Fig. 1A represents one subunit consisting of a CI-CII pair). KaiA binds to the CII domain of KaiC, which stimulates autophosphorylation^22,23^. Phosphorylation accumulates slowly throughout the day, first on Thr432, then on Ser431. When Ser431 is heavily phosphorylated (shown in Fig. 1B), ring-ring stacking interactions allow the CII domain to regulate the slow ATPase cycle in CI^24^. The post-hydrolysis state of CI allows KaiB to bind to KaiC^25^ and six KaiB molecules to assemble cooperatively on the CI ring^26,27^.

This KaiB-KaiC complex sequesters and inactivates KaiA, closing a negative feedback loop to inhibit further phosphorylation and allowing KaiC to dephosphorylate. Unphosphorylated KaiC then releases KaiB and is ready to begin the cycle again. The kinetics of the phosphorylation rhythms are remarkably robust to temperature and protein concentration, yet can be tuned dramatically by single amino acid substitutions^28,29^. The system is also remarkably thrifty in its energy consumption—with each KaiC molecule consuming only 15 ATP per day^30^. Thus, the Kai protein system is a uniquely attractive choice to develop into a synthetic tool to endow materials with programmable, autonomously time-dependent properties.

## Results and Discussion

### Biotinylation of KaiB allows KaiC to mediate material crosslinking.

**Figure 1.**
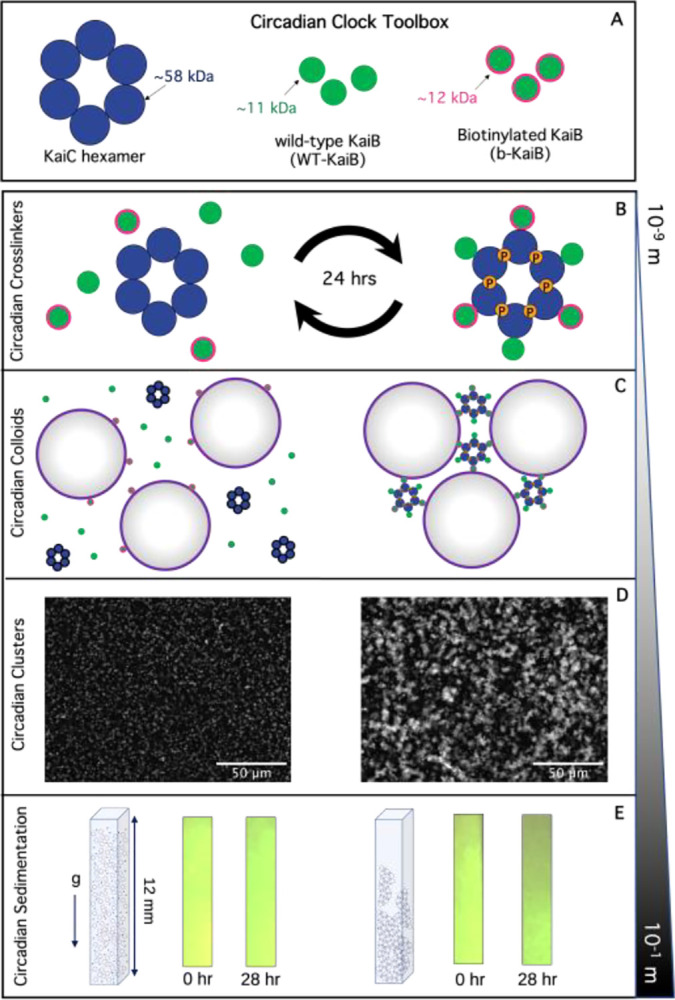
Harnessing circadian clocks to engineer non-equilibrium materials across scales. **(A)** We functionalize cyanobacteria clock proteins to couple to materials by biotinylating KaiB (b-KaiB) and incorporating b-KaiB (green with magenta outlines) into the clock system comprising KaiB (green), hexameric KaiC rings (blue), and KaiA (not shown). **(B)** KaiB monomers bind cooperatively to KaiC rings in a phosphorylation-dependent manner (indicated by the orange ‘P’ circles) and are subsequently released as KaiC dephosphorylates over a 24-hr cycle. We exploit the transition from free KaiB to KaiB fully assembled on a KaiC hexamer to create a time-dependent and phosphorylation-dependent change in crosslinking valency. **(C)** We incorporate the ‘circadian crosslinkers’ depicted in (B) into suspensions of 1-μm streptavidin-coated colloids to drive time-dependent crosslinking of colloids. **(D)** Microscope images of fluorescent streptavidin-coated colloids, mixed with KaiB, b-KaiB, and KaiC phosphorylation site mutants that cannot bind KaiB (pT KaiC, left) or constitutively bind KaiB (pS KaiC, right), show that KaiB-KaiC assembly selectively causes mesoscale clustering and connectivity of colloids. **(E)** Sedimentation of circadian colloidal suspensions shown in (D) demonstrate pronounced settling of colloids after a day of incubation with the mutant that forms KaiB-KaiC complexes (right) compared to colloids mixed with the non-binding KaiC mutant (left).

To harness the Kai protein system for materials activation, we aimed to exploit the changing oligomeric state of KaiB, induced by interaction with KaiC, throughout the circadian cycle. Namely, KaiB transitions from being free in solution to forming a hexameric KaiB-KaiC complex (KaiBC). We reasoned that functionalized KaiB molecules would not be effective crosslinkers of material components when KaiB is free in solution, but that assembly into the KaiBC complex might create a potent multivalent crosslinker (Fig. 1B). To develop this tool and characterize its effect, we chose a commercial colloidal suspension as a model material platform (Fig. 1C). We hypothesized that as the number of colloids able to participate in KaiC-mediated crosslinks increased, via increasing KaiBC assembly, we would observe a transition from a fluid-like suspension of single colloids, to a gel-like state with larger connected clusters of colloids (Fig. 1D). Consistent with this prediction, we observe macroscopic changes in the ability of the colloidal material to sediment (Fig. 1E).

**Figure 2.**
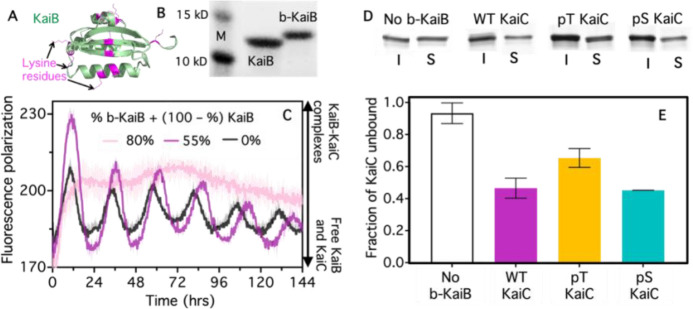
Biotinylation of KaiB preserves oscillator function and phosphorylation-dependent binding to KaiC. **(A)** Potential sites of amine-reactive biotinylation (lysine residues in magenta) overlaid on the KaiB crystal structure (green)^31,32^. **(B)** SDS-PAGE gel of unlabeled KaiB and biotinylated KaiB (b-KaiB), shows successful biotinylation indicated by a mobility shift of the biotinylated product (molecular weight standards (M) are 10 kDa and 15 kDa). **(C)** KaiABC reactions in the presence of biotinylated KaiB. Oscillations are measured by fluorescence polarization of FITC-labeled KaiB (0.2 μM), a read-out of KaiB-KaiC complex formation. All conditions contain 3.5 μM KaiB, with the specified fraction being b-KaiB. Oscillatory association of KaiB with KaiC is sustained with 55% b-KaiB (magenta), the percentage used in subsequent experiments, but not with 80% (pink). Each curve is an average of two replicates. **(D,E)** Streptavidin pull-down analysis of the KaiB-KaiC interaction. Data from left to right are for: wild-type KaiC with 100% KaiB (No b-KaiB, white), wild-type KaiC with 55% b-KaiB (WT KaiC, magenta), non-binding pT KaiC with 55% b-KaiB (pT KaiC, yellow), and constitutively-binding pS KaiC with 55% b-KaiB (pS KaiC, cyan). **(D)** SDS-PAGE gel of KaiC remaining in the supernatant (S), and thus not in complex with b-KaiB, following pull-down using magnetic streptavidin-coated beads. Each pair of bands corresponds to KaiC in the input reaction after 8-hr incubation (I) and supernatant following pull-down (S). **(E)** Ratios of the supernatant and input determined by gel densitometry for each pair shown in (D), with 0 indicating that all KaiC is bound to b-KaiC while 1 indicates no KaiC is bound.

To endow the KaiBC complex with time-dependent material crosslinking properties, we first needed to functionalize KaiB to bind to the colloids strongly and statically, which we achieved through biotinylation of KaiB (Fig. 2A,B) and the use of streptavidin-coated colloids. We next needed to ensure that oscillatory KaiBC complex assembly was preserved when incorporating biotinylated KaiB (b-KaiB), and that b-KaiB could still bind to KaiC in a phosphorylation-dependent manner. To achieve the former, we used a fluorescence polarization assay to monitor rhythmic complex formation in the KaiABC reaction, finding that the reaction could tolerate at least half of the KaiB proteins being replaced by b-KaiB while still producing high amplitude rhythms (Fig. 2C). To quantify the ability of KaiC to bind to b-KaiB, we performed pull-down experiments with b-KaiB-coated magnetic beads (see SI Methods) and measured the fractions of KaiC that remained in the supernatant (not bound to b-KaiB-beads). Fig. 2D,E shows that b-KaiB retains its ability to interact with KaiC, resulting in less KaiC in the supernatant (S) compared to the input (I) (Fig. 2D), as well as its preference for the pS431 state (pS) of KaiC compared to pT432 (pT) (Fig. 2E).

**Figure 3.**
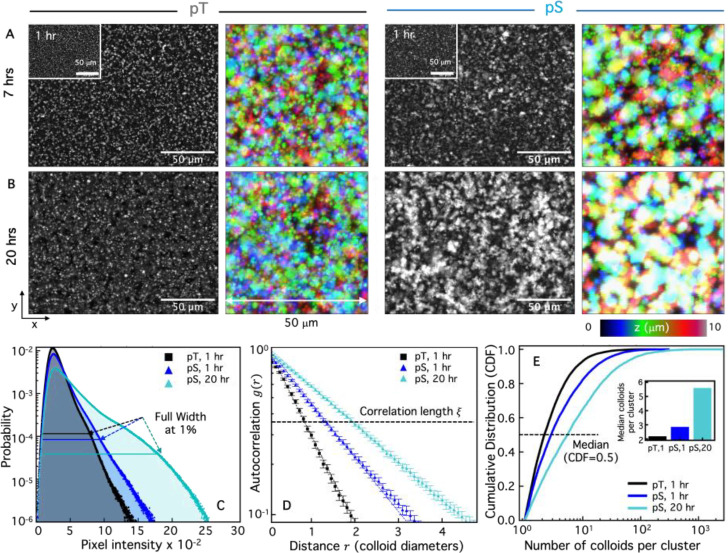
pS KaiC selectively mediates clustering of colloids quantified by complementary image analysis methods. **(A,B)** Greyscale microscope images and colorized *z*-projections of fluorescent colloids taken at 1 hr (A insets), 7 hrs **(A)** and 20 hrs **(B)** after mixing with KaiC mutants that are frozen in non-binding (pT, left subpanels) or binding (pS, right subpanels) states. Epifluorescence microscope images and *z*-projections of confocal image stacks show substantial clustering and assembly of pS-colloids over time that is absent for pT-colloids. **(C-E)** Quantification of colloidal clustering in epifluorescence images by evaluating **(C)** the pixel intensity distributions of images, **(D)** spatial image autocorrelation (SIA) function *g*(*r*), i.e., the correlation between two pixels separated by a radial distance *r*, and **(E)** the normalized cumulative distribution (CDF) of cluster sizes, where a cluster is defined as a connected set of above-threshold pixels. To compare these complementary analyses, we compute **(C)** the full width of each intensity distribution at 1% of the corresponding mode, denoted by the horizontal lines; **(D)** the characteristic correlation length ξ, determined by fitting each *g*(*r*) curve to an exponential function (dashed lines), and denoted by the corresponding intersection of the horizontal line at *g* = *e*^−1^; **(E)** the cluster size at CDF = 0.5, i.e., the median cluster size, as denoted by the intersection of the horizontal line with each CDF. The data shown in C-E are from images collected at 1 hr for pS (black) and pT (blue) and 20 hrs for pS (cyan). Further details regarding the analyses depicted in C-E are described in Methods and SI.

We next aimed to test the ability of KaiC to selectively crosslink b-KaiB-coated colloids via KaiBC complex assembly. To this end, we mixed b-KaiB into a suspension of 1-μm diameter streptavidin-coated colloids, then added either the pS KaiC mutant or the pT KaiC mutant to the suspension. pS and pT are mutated at the phosphorylation sites of KaiC to mimic either a state that permanently allows KaiB binding (pS—S431E;T432A) or prevents binding (pT—S431A;T432E). By imaging the fluorescent-labeled colloids, we found that the colloids remained largely as isolated microspheres in the presence of the non-binding pT mutant, exhibiting no preferential self-association even after a day of incubation (Fig. 3A,B). Corresponding confocal z-stacks show that the colloids are isotropically dispersed throughout the 3D sample, as seen by the small features and lack of white regions in the color-coded *z*-projection of representative image stacks (Fig. 3A,B). This minimal self-association is similar to that observed without b-KaiB (Fig. S1), indicating that non-specific crosslinking is low. In contrast, Fig. 3A shows that b-KaiB-coated colloids incubated with the binding-competent pS KaiC mutant grow into large colloidal aggregates, ultimately assembling into super-structures that extend across the height of the flow cell channel, as seen by the large white regions of the color-coded *z*-projections (Fig. 3B).

Microscopy images qualitatively demonstrate the efficacy and selectivity of KaiBC-mediated crosslinking (Fig 3A,B). To quantify the nature and extent of the observed clustering, we developed multiple quantitative metrics to fully capture the spatiotemporal diversity of the crosslinked structures we observe in the experiments (Fig 3C–E).

We first evaluated the probability distribution of pixel intensities in the images. In the fluorescent images, the crosslinked beads overlap to make larger, brighter regions of pixels. Larger clusters are brighter from contributions from parts of the colloidal cluster above and below the focal plane. As we would expect from the images shown in Fig. 3A,B, the distribution for pS KaiC after 20 hrs has a pronounced tail of high intensity pixels values. The distributions for pT and pS after 1 hr are substantially narrower (Fig. 3C), suggestive of minimal structural heterogeneity and clustering. As a proxy for the degree of clustering, we compute the full width of each distribution at 1% of its peak (mode) probability (FW1%). The FW1% metric is larger for samples with larger clusters.

In a second approach, we use spatial image autocorrelation (SIA) Fourier analysis to quantify the correlation *g*(*r*) between pixels separated by a radial distance *r* (Fig. 3D). *g*(*r*) decays exponentially from *g*(0) = 1 with the decay rate indicating the characteristic size of features in an image. Slower decay of *g*(*r*) with *r* indicates larger features (i.e. clusters), as seen for pS compared to pT and long compared to short times. By fitting each *g*(*r*) curve to an exponential decay, we quantify a characteristic correlation lengthscale *ξ* of the colloidal system, indicated by the distance *r* at which the dashed horizontal line intersects each curve in Fig. 3D. It is clear that the correlation length is larger for the constitutively binding pS KaiC both at 1 hr and even larger at 20 hrs (Fig. 3D).

Finally, we directly detected the number and size of clusters of colloids in the imaging plane by binarizing the images and computing the sizes of the connected regions of pixels that have intensity above a specific threshold (see SI Methods). Evaluating the cumulative distribution function (CDF) of cluster sizes, we observe an increase in clustering in the pS samples. The enhanced clustering is apparent both as an increase in the median size of clusters, and the emergence of a long tail in the distribution of cluster sizes, especially at 20 hrs (Fig 3E). These quantitative metrics show clear differences for the non-binding pT and constitutively-binding pS mutants, giving us confidence that they are robust methods to evaluate the kinetics and phosphorylation dependence of KaiBC-mediated self-assembly of colloidal clusters.

### Crosslinking proceeds with circadian kinetics.

**Figure 4.**
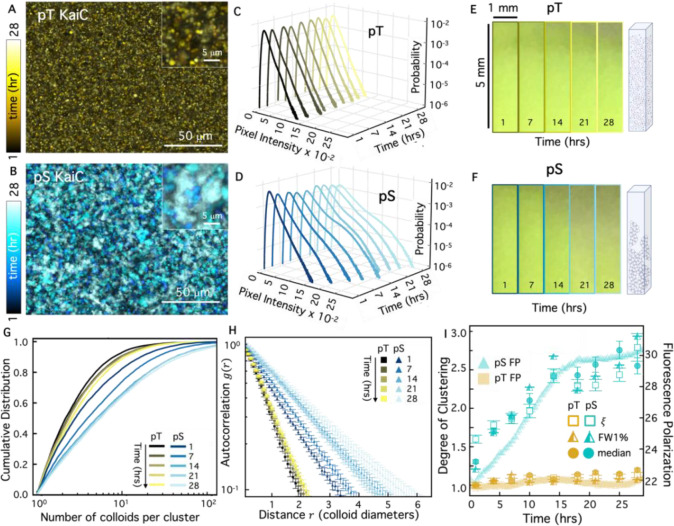
Time-dependence of KaiC-mediated cluster formation. **(A,B)** Colorized temporal projections of time-lapses of pT (A, yellow) and pS (B, cyan) over the course of 28 hrs, with colors indicating increasing time from dark to light according to the colorscales. Zoomed-in insets show different degrees of clustering over time for different KaiC mutants. **(C,D)** Pixel intensity probability distributions for pT (C, yellow), and pS (D, cyan) at different times over 28 hrs, with lighter shades denoting later times according to the color scales in A,B. Distributions show broadening and emergence of high-intensity peaks at later times for pS. **(E,F)** Images of a colloidal suspension undergoing sedimentation in a capillary (dimensions listed) over 28 hrs in the presence of pT (E, top) and pS (F, bottom). Images show that pS-colloids sediment more quickly, as indicated by dark regions extending further down the images. The time that each image is captured is listed at the bottom, and the cartoons to the right of panels respectively depict the expected state of the suspension (not drawn to scale). **(G,H)** CDFs of cluster sizes (G) and *g*(*r*) curves (H) for 5 different times between 1 and 28 hrs for pT (yellow) and pS (cyan) with color shade indicating time according to the legends in A,B. **(I)** Correlation lengths *ξ* (open squares), full width at 1% max (half-filled triangles), and median cluster size (filled circles), each normalized by their initial pT value, show that the time-course of cluster assembly over 28 hrs for pT (gold) and pS (cyan) correlate with the fluorescence polarization (FP) of KaiB (right axis, arbitrary units), which serves as a proxy for KaiBC complex formation. Both the degree of clustering and FP remain at a minimum for pT, while for pS, both steadily increase for the first ~15 hrs.

To characterize the circadian clustering kinetics that drive the colloidal suspensions from comprising freely floating particles (Fig. 3A) to large, connected super-structures (Fig. 3B), we collected images of the emerging clusters at nine different time-points over the course of a day. To promote mixing and limit colloid settling and sticking, we kept all samples under continuous rotation between imaging intervals. Overlaying temporally color-coded images from these time-course experiments confirms that structure emerges over time in the pS KaiC sample (Fig. 4B), while minimal clustering is seen in the presence of pT (Fig. 4A). The corresponding pixel intensity distributions (Fig. 4C,D) likewise show narrow distributions for pT that do not change significantly over time, while pS distributions display gradually elongating high-intensity tails and emerging multi-modal curvature. We also note that pS distributions appear to broaden more substantially in the first half of the experiment (~15 hrs) compared to the second half. To demonstrate that these mesoscopic structural changes can translate to macroscopic material changes, we imaged colloidal suspensions undergoing sedimentation in capillaries over the course of a day (Fig. 4E–F). The larger clustering seen for pS is mirrored by pronounced sedimentation of the suspension, while the pT colloids remain suspended.

The slow KaiC-dependent cluster assembly is also evidenced by the time-evolution of the cluster size CDFs (Fig. 4G) and autocorrelation curves *g*(*r*) (Fig. 4H) which both show that pS KaiC samples form clusters that continue to grow over time. These data also indicate that, unlike the pT sample, some clustering has already occurred in the pS sample at the first time point (*t* = 1 hr). However, the emergence of more clusters and growth of existing clusters continue over the course of incubation.

Given that pS KaiC is locked into a binding-competent state, the gradual self-assembly of colloids over many hours of incubation suggests that the rate-limiting step in self-assembly is the complexing of KaiB and KaiC. Indeed, KaiBC complexes are known to form on the timescale of many hours, likely due to both the slow ATPase cycle in the KaiC CI domain^33^ and the time required for KaiB to refold into an alternative fold-switched structure^34,35^. To test this hypothesis, we measured the kinetics of the KaiBC interaction using fluorescence polarization and compared to the kinetics of material self-assembly. Specifically, in Fig. 4I, we plot the time evolution of the relative fluorescence polarization (FP), which reports on formation of KaiBC complexes, with the metrics of cluster formation that we determine from the data shown in Fig. 4C,D (FW1%), G (median cluster size), and H (correlation length ξ). To compare these different clustering metrics, we normalize each quantity by the corresponding initial value for pT such that the values indicate the degree of clustering, which is 1 in the absence of clusters. As shown, the KaiBC interaction kinetics grow approximately linearly for the first 15 hours after which they approach saturation, likely reflecting a regime where the majority of both KaiB and KaiC molecules are in complex and have been depleted from solution. The degree of clustering follows a similar time-course, with a shift at ~15 hrs to slower, asymptotic cluster formation. This agreement between the kinetics of KaiBC interactions and material self-assembly strongly suggests that the biochemical properties of the Kai proteins, such as the KaiC catalytic cycle, are regulating the rate of cluster growth.

### Simulations with multiple bonds per particle recapitulate timing and irreversibility of cluster formation.

**Figure 5.**
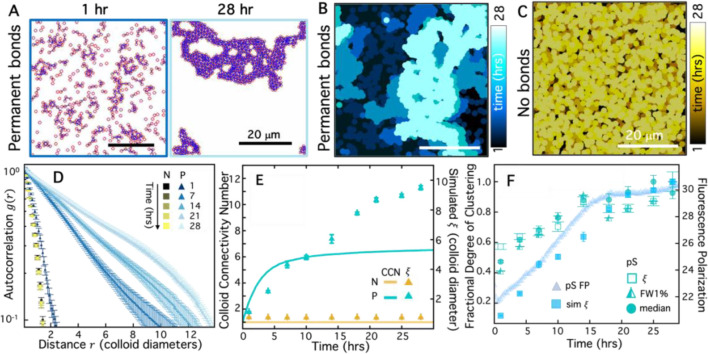
Simulations of circadian colloids recapitulate experimental results and reveal the necessity for multiple bonds. **(A)** Simulation snapshots showing clustering of colloids (red circles) crosslinked by permanent bonds (purple lines), analogous to the experimental pS-colloid system, at 1 hr (left) and 28 (right) hrs. **(B,C)** Colorized temporal projections of simulation snapshots for colloids (filled circles) with permanent crosslinker bonds (B, cyan), analogous to pS, and colloids with no crosslinker bonds (C, yellow), analogous to pT. Times of simulation snapshots used in the projections are the same as in Fig. 4A.B, with colorscales indicating increasing time from dark to light. **(D)** *g*(*r*) versus radial distance *r* computed for simulation snapshots, taken at times specified in the legend, for colloids with No bonds (N, yellow squares) and Permanent bonds (P, cyan triangles). **(E)** Time-course of the colloid connectivity number CCN (left axis, solid lines) and correlation lengths *ξ* (right axis, triangles) determined from simulations with Permanent bonds (P, cyan) and No bonds (N, gold). **(F)** Multiple metrics of clustering and self-assembly resulting from permanent crosslinker bonding in experiments (pS) and simulations (P), each normalized by its maximum value to indicate the fractional degree of clustering (left axis) measured by each data type. Metrics include: experimental correlation lengths (𝜉, open squares), simulated correlation lengths (sim ξ, filled squares), full width at 1% (FW1%, half-filled triangles), and median cluster size (median, filled circles). Trends in both simulation and experimental data track with the time-course of KaiB fluorescence polarization (right axis, translucent triangles) in a reaction with pS KaiC.

The correlation of the KaiB fluorescence polarization with the clustering of colloids suggests that the Kai activity is driving the kinetic control of clustering. In order to assess this mechanism, we developed a numerical simulation that captures the key components of our experimental system (see Methods and SI). In the simulations, 1-μm diameter colloids move via Brownian motion in a 50 μm × 50 μm 2D plane, and, when the surfaces of two colloids are within 10 nm of each other (comparable to the size of the KaiBC complex^36^), they can form a bond mediated by b-KaiBC complexes. KaiB and KaiC are assumed to be present at constant concentrations and their interaction to form crosslinks is treated phenomenologically. The probability of complex formation during an encounter is a constant value chosen to match the solution binding kinetics (see SI). We allow simulations to run for 30 hrs and capture the state of the colloids at the same time intervals as in experiments (Fig. 5A).

To model our experimental pS KaiC and pT KaiC colloidal systems (Fig. 4), we consider cases in in which, respectively, bonds between colloids are incapable of releasing once they are formed (Permanent bonds, Fig. 5A–B) and bond formation probability is zero (No bonds, Fig. 5C). The color-coded temporal overlays of simulation images with permanent bonds (P) and no bonds (N) show qualitative similarities with the experimental overlays of pS (Fig. 4B) and pT (Fig. 4A). However, the simulated clusters appear to be larger than the experimentally observed clusters, which we may expect because the simulated colloids are confined to grow in two dimensions (in the image plane), whereas confocal *z*-stacks (Fig. 3B) and pixel intensity distributions (Fig. 4D) of experimental pS images suggest that colloidal clusters also grow and extend perpendicular to the imaging plane. To quantitatively compare simulated and experimental cluster assembly kinetics, we perform the same SIA analysis that we use for experimental images (Fig. 3D, 4H) to compute time-dependent autocorrelation curves (Fig. 5D) and corresponding correlation lengths (Fig. 5E). Similar to the *g*(*r*) trends we observe for experimental pT and pS images (Fig. 4H), Fig. 5D shows that *g*(*r*) for the ‘No bonds’ system (N) exhibits minimal time-dependence and fast decay with distance *r*, indicative of small features that do not change size over time. Conversely, *g*(*r*) for the ‘Permanent bonds’ case decays more slowly than N at all time-points and broadens substantially over time, indicative of larger clusters that grow over time. The time-course of the corresponding correlation lengths of the 30-hr simulation are likewise similar to the experimental trends in Fig. 4I, with *ξ* values for the N case growing over time and transitioning to slower increase in the latter half of the simulation.

The continued cluster growth for pS (experiments) and P (simulations) is somewhat unexpected given the saturation of the fluorescence polarization at ~15 hrs. Specifically, FP saturation suggests that all possible KaiBC complexes have formed, while the colloid data suggest that clusters continue to form and grow after this saturation. To shed light on this seeming paradox, we compute the colloid connectivity number (CCN) from simulations, which measures how many neighboring colloids are connected to a single colloid. Because of the 2D geometry and the size of the colloids, the maximum possible CCN is six. Fig. 5E shows that the CCN increases to saturating levels over the course of ~10–15 hrs, similar to the KaiBC FP data, while the simulated correlation lengths continue to increase after this time, albeit less dramatically than the first half of the time-course. These data indicate that the rate-limiting step in colloid crosslinking is the assembly of KaiBC complexes rather than the time needed for colloids to come into close contact.

Our results further indicate that cluster growth can proceed even when the majority of colloids are saturated with permanent crosslinks. Such assembly kinetics may arise if the majority of colloids are on the interiors of clusters and saturated, while those on the boundaries may have available b-KaiB binding sites to crosslink to other colloids on the edges of neighboring clusters. Self-assembly thus transitions from that of single colloids coming together to form clusters, to one in which most colloids are participating in clusters that then merge to form larger super-structures. Fig. 5F corroborates this physical picture by comparing the kinetics of cluster formation in the experimental and simulation data with the KaiBC assembly kinetics. The similarity in the shapes of the experimental and simulation curves suggests that the model is indeed capturing the underlying process generating clusters. The clear shift in kinetics at ~15 hrs in all data further corroborate the robustness of the simulations, and demonstrate that self-assembly is rate-limited by the timescale of KaiBC complex formation.

### Oscillations in circadian protein interactions lead to sustained assembly of colloidal networks.

**Figure 6.**
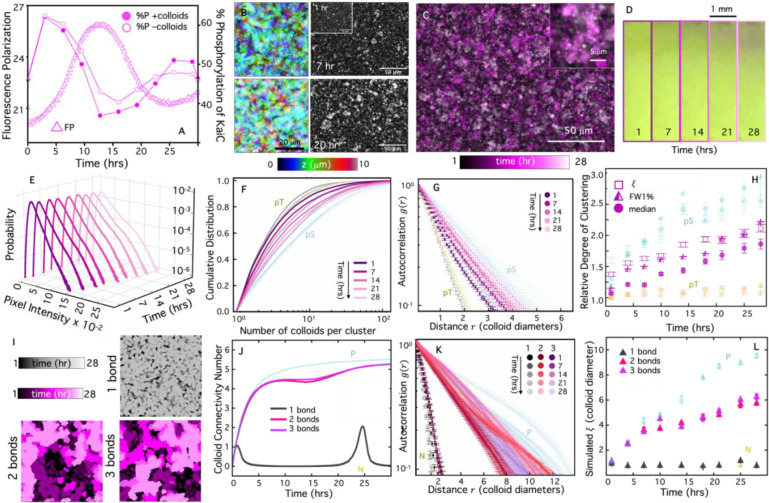
The time-course of colloidal clustering is dictated by the oscillating phosphorylation state of wild-type KaiC and the KaiBC bonding valency. **(A)** Fluorescence polarization (FP) of KaiB (left axis, triangles) and percentage of phosphorylated KaiCs (%P, right axis, circles) during a KaiB-KaiC reaction. %P measurements were performed in the presence (filled circles) and absence (open circles) of colloids, showing that KaiC phosphorylation dynamics are unaffected by the presence of colloids. **(B)** Colorized *z*-projections (left) and greyscale microscope images of fluorescent colloids taken at 1 hr (inset), 7 hrs (top) and 20 hrs (bottom) after mixing with wild-type KaiC. Image properties and acquisition parameters are as in Fig. 3A,B. **(C)** Colorized temporal projection of KaiC images over the course of 28 hrs, with zoomed-in inset, shows clustering over time. Colors shades indicate time according to the colorscale, and image properties and acquisition parameters are as in Fig. 4A,B. **(D)** Images of a colloidal suspension undergoing sedimentation under identical conditions as in B and C. **(E)** Corresponding pixel intensity probability distributions for different times over 28 hrs, denoted by the colorscales in A,B. **(F,G)** Cluster size CDFs (F) and autocorrelations *g*(*r*) (G) for wild-type KaiC (magenta shades) at 5 different times between 1 and 28 hrs, indicated by color shade. **(H)** (left axis) Experimental metrics of clustering, each normalized by its initial pT value, indicate the relative degree of clustering above a baseline value of 1. Metrics include: correlation length (ξ, open squares), full width at 1% (FW1%, half-filled triangles), and median cluster size (median, filled circles). **(I)** Colorized temporal projections of snapshots captured from simulations that mimic 1 (top, grey), 2 (bottom left) or 3 (bottom right) oscillatory crosslinkers per bead connection. **(J)** CCN versus time for simulations with 1 (grey), 2 (magenta) or 3 (lilac) bonds per colloid connection. **(K)** *g*(*r*) versus radial distance *r* (in units of colloid diameter) measured at various time-points during simulations with 1, 2 and 3 bonds, as indicated by color shades according to the legend. **(L)** Time-course of correlation lengths *ξ* for simulations with 1 (grey), 2 (magenta) and 3 (lilac) crosslinker per beads connection. In (F)-(H) and (J)-(L), translucent pT or N (yellow) and pS or P (cyan) data, previously shown in Fig. 4 or 5, are shown for comparison to the oscillating cases.

Having demonstrated that material self-assembly can be temporally programmed by the phosphorylation state of the circadian clock proteins, we now investigate the effect of oscillatory interactions between KaiB and KaiC on the colloid connectivity and cluster assembly kinetics. To achieve oscillatory crosslinking, we replace the phosphorylation-locked mutants with WT KaiC and add KaiA, creating a circadian rhythm in both KaiC phosphorylation (mediated by KaiA) and the KaiB-KaiC interaction (Fig. 1B). We first aimed to demonstrate that the ~24 hr oscillation in in KaiBC complex formation is not disrupted by the presence of colloids using the protein concentrations, buffer conditions, and 1.26% colloid volume fraction used in the mutant KaiC experiments (Figs. 3,4). Fig. 6A confirms that the expected oscillation in KaiBC complexing, measured by fluorescence polarization, is unperturbed; and that the oscillatory phosphorylation of KaiC is similarly preserved and largely unaffected by the presence of streptavidin-coated colloids.

To map the effect of oscillating crosslinkers onto autonomous material behavior, we perform the same microscopy experiments (Fig. 6B,C) and analysis (Fig. 6E–H) as with the pS and pT mutants. As shown in Fig. 6B–C, colloids indeed form clusters during the binding phase of the rhythm, as we expect. However, surprisingly, the clusters do not noticeably disassemble during the subsequent unbinding phase. Moreover, the clusters appear smaller and more globular in shape than the extended super-structures formed in the presence of the constitutively binding pS mutant. Similarly, at the macroscopic scale, we observe sedimentation over the course of a day that is substantially more pronounced than for non-binding pT KaiC but less stark than with pS (Fig. 6D).

Using our quantitative metrics (Fig. 3C–E), we evaluate the corresponding colloidal self-assembly kinetics (Fig. 6E–G). The pixel intensity distributions steadily broaden over time with a secondary high-intensity population emerging, indicative of cluster formation and growth (Fig. 6E). The corresponding cluster size distributions (Fig. 6F) and autocorrelation curves (Fig. 6G) corroborate these findings and show that cluster sizes and correlation lengthscales grow over time for the oscillatory case, starting out larger than the non-binding pT case but remaining lower than the pS case for all time-points during the oscillatory cycle. Fig. 6H, which compares the time-course of the metrics computed from each analysis method (Fig. 6E–G), shows that the Kai oscillator increases the degree of clustering over the full oscillatory cycle, but the degree of clustering remains lower than that of the pS mutant, in particular at later times in the cycle. Moreover, the growth appears to flatten during the ~10–20 hr trough of the phosphorylation cycle (Fig. 6A).

To understand how oscillating crosslinkers can drive monotonic material self-assembly, we build on the simulations described above to allow colloid binding and unbinding rates to vary sinusoidally throughout the reaction (see Methods, SI). In brief, we consider the same binding rate amplitude $p_{o}$ as in the permanent bond case but we incorporate an oscillation of this rate, $p_{on}=p_{o}\cos^{2}(\pi t/T)$, where T is the oscillation period. We also add a dissociation rate with the same amplitude and functional form as the binding rate, but that is π/2 radians out of phase, i.e., $p_{d}=p_{o}\sin^{2}(\pi t/T)$. Within this framework, if we treat each connection between two colloids, quantified by CCN, as formed by a single KaiBC complex, we observe minimal cluster formation or structural time-dependence, as evidenced by the color-coded temporal collapse of the simulated images for the ‘1 bond’ case shown in Fig. 6I.

However, given the saturating level of Kai proteins in our experiments (~10^5^ b-KaiB proteins per colloid) and the two orders of magnitude smaller size of the crosslinkers compared to the colloid surface area, it is highly likely that multiple KaiBC complexes participate in a single connection between two colloids. To simulate this effect, we consider cases in which each colloid connection is mediated by either 2 or 3 KaiBC crosslinker bonds (Fig. 6I). In both cases, clusters form and grow over time with features that are quite similar to the temporal collapse of the experimental oscillatory data (see Fig. 6C inset).

The similarity between the ‘2 bond’ and ‘3 bond’ cases, and their dramatic difference from ‘1 bond’, suggest similar variation in the kinetics of colloid connectivity for single versus multiple bond cases. This conjectured behavior can be seen clearly in the time-course of the CCN for the three cases (Fig. 6J). When only a single KaiBC-mediated bond connects two colloids, oscillatory connectivity is evident, with peaks in CCN observed at times that roughly correlate with the measured peaks in KaiC phosphorylation. However, the peak CCN values of ~1–2 are low compared to saturation and to the permanent bond case, and non-zero CCN values are only maintained for a small fraction of the oscillation period, preventing large structures from forming and remaining assembled. The situation is quite different for the multiple bond case, in which colloid connectivity increases rapidly at early times, at a similar rate as for the Permanent bond case, and does not dissolve during the disassembly phase. Rather, each colloid loses only a small fraction of connections during the KaiBC dissolution phase and approaches saturating levels during the second phosphorylation peak.

By comparing *g*(*r*) for the simulations with different bonding valencies at various time points during the circadian cycle (Fig. 6K), we find that the ‘1 bond’ case exhibits unnoticeable time-dependence and minimal clustering, similar to the simulated No bonds (N) case and experimental pT case (Fig. 6G). On the other hand, *g*(*r*) for both multivalent cases mirror the experimental trends measured for the oscillatory KaiBC system (compare Fig. 6G and 6K), broadening over time but continuing to decay more quickly than the Permanent bond (P) case at all time-points. The time-dependent correlation lengths computed from each *g*(*r*) for the different valencies, which quantify the assembly kinetics, follow an analogous time-course as the experimentally measured clustering metrics for the oscillatory case shown in Fig. 6H, suggesting the need for multivalent bonding to sustain assembly. At the same time, the stochastic nature of the KaiBC binding limits the ability of colloids to undergo oscillations in cluster formation as cluster dissolution would require unbinding of the majority of crosslinkers. Intuitively, a large, heavily crosslinked cluster will likely not dissolve unless the vast majority of bonds are broken.

## Outlook

Biomolecular signaling systems typically must maintain their function with high fidelity while interacting with numerous other components crowded within living cells and while subject to unpredictable fluctuations in their environment. These constraints equip networks of interacting biomolecules with unique robustness properties that may allow them to be harnessed to endow synthetic materials and systems with functionality, programmability and autonomous reconfigurability. However, coupling biomolecular systems to synthetic materials to impart desired properties remains a grand challenge in active matter and biomaterials research^13,37–40^. Here, we break new ground by using the KaiABC circadian oscillator as a prototypical example of a robust biomolecular signaling system. We demonstrate that this system can tolerate being functionalized to act as a material crosslinker, and that it can then be used to autonomously regulate timed material self-assembly. Specifically, we show that Kai proteins can assemble colloidal suspensions into networks of mesoscopic clusters at rates and efficiencies that are controlled by the phosphorylation state of KaiC. These molecular interactions translate to bulk changes in the sedimentation properties of the materials, visible by the naked eye. Moreover, our mathematical models show that the valency of circadian crosslinkers can be used as a switch to allow either sustained self-assembly or rapid dissolution of the material.

This proof-of-concept opens the door to Kai-mediated oscillatory crosslinking of a diversity of synthetic and natural materials, such as hydrogels, polymeric fluids, cellulose, and granular materials, to drive user-defined autonomous changes in material properties. These designs can be used to create technologies such as dynamic filtration and sequestration devices, self-healing infrastructure, and programmable wound suturing. Beyond material crosslinking, the KaiBC system could be used as a synthetic scaffold to gate enzymatic activity to control the release of drugs or achieve metabolic channeling by enforcing spatial proximity between other entities. Beyond oscillation, biological systems are capable of many information processing tasks including thresholding, fold-change detection, and sign-sensitive filtering of input signals. Because these systems all function based on high molecular specificity, they represent a natural library of computational devices that can be coupled to non-biological systems to achieve autonomous control.

## Methods

Complete methods and materials are provided in the Supplementary Information. Key details are provided below.

### Protein preparation and characterization:

KaiA, KaiB, KaiC, pT KaiC (KaiC-AE; S431A, T432E), and pS KaiC (KaiC-EA; S431E, T432A) were recombinantly expressed and purified as previously described^33,41^. Purified proteins were buffer-exchanged into Kai buffer containing 10% glycerol, 150 mM NaCl, 20 mM Tris-HCl (pH 8.0), 5 mM MgCl_2_, 0.5 mM EDTA (pH 8.0), and 1 mM ATP (pH 8.0). KaiB was functionalized with biotin (b-KaiB) using EZ-Link-Sulfo-NHS-LC-Biotin (ThermoFisher) at a 50× molar excess to KaiB (Fig. 2A–C). The pull-down assay to assess specific binding of KaiC to b-KaiB (see Fig. 2D,E) included 6.5 μM KaiC (wild-type, pT mutant or pS mutant) and 5.5 μM KaiB (55% b-KaiB, 45% unlabeled KaiB) in Kai buffer. Following 8-hr incubation at 30°C, b-KaiB and its binding partners were removed from solution using streptavidin-coated magnetic beads (Cytiva). The resulting supernatant was analyzed by SDS-PAGE (Fig. 2D,E).

To characterize the tolerance of the standard oscillator reaction to b-KaiB, we measured the fluorescence polarization of KaiB in reactions with 1.5 μM KaiA, 3.5 μM KaiC, different ratios of KaiB and b-KaiB, and 0.2 μM FITC-labeled KaiB^K25C^ over the course of 144 hrs, similar to the procedure used previously^42^. We performed the same assay to characterize protein function under colloid-linking conditions which include 5.5 μM KaiB (55% b-KaiB), 6.5 μM KaiC (wild-type, pT or pS), 2.2 μM KaiA, and 0.4 μM FITC-labeled KaiB^K25C^. To characterize the phosphorylation state of KaiC in the colloidal system, KaiC phosphoform composition was resolved by SDS-PAGE analysis. The ratio of phosphorylated KaiC to unphosphorylated KaiC was quantified by gel densitometry.

### Circadian colloid experiments:

We used 1.0-μm diameter streptavidin-coated polystyrene microspheres (Fluoresbrite YG Polysciences) as the colloids in all experiments. Colloids were washed and resuspended in Kai buffer such that the final concentration in all experiments is 1.26% solids (~4.0×10^−5^ μM). To prepare Kai-colloid suspensions, we add 3.6 μM b-KaiB, 2.9 μM wild-type KaiB, 2.2 μM KaiA, and 6.5 μM of either wild-type KaiC, pS KaiC or pT KaiC. The time of KaiC addition sets *t* = 0 for each experiment. For microscopy experiments, Kai-colloid suspensions were flowed via capillary action into passivated sample chambers consisting of a glass microscope slide and No. 1 coverslip fused together with heated ~120-μm thick parafilm spacers to accommodate 8 μL of sample. After sealing the chambers with UV glue, they were immediately placed on a 360° rotator at 30°C for the duration of each ~30 hr experiment except when being imaged. For each experiment two replicates were prepared and imaged immediately after one another.

Colloidal suspensions were imaged using an Olympus IX73 epifluorescence microscope with a 40× 0.6 NA objective, 480/535-nm excitation/emission filters, and a Hamamatsu ORCA-Flash 2.8 CMOS camera. For each condition and time-point, 6 images were captured in equidistant regions in the sample chamber within a span of 5 mins. All data shown consists of 2–3 replicate experiments with vertical error bars representing standard error. To construct confocal *z*-stacks (see Fig. 3, 6), we used a Nikon A1R scanning confocal microscope with a 60× 1.4 NA oil-immersion objective, a 488 nm laser and 488/595 nm excitation/emission filters. Stacks of 51 images were constructed using a 0.2 μm *z*-step size for a total height of *z* = 10 μm.

We processed and analyzed images, as depicted in Fig. 3, using custom-written Python codes^43–45^. We evaluated the distribution of pixel intensities across all images for a given time and condition, which we normalize to probability density distributions. We performed spatial image autocorrelation (SIA) analysis in Fourier space and directly measured 2D cluster sizes in real space. For both analyses, we first binarized images using local thresholding algorithms^46^. To measure cluster sizes, we identified each connected set of pixels above threshold as a cluster, counted the number of pixels in each such region, and divided by the cross-sectional area of a colloid. To quantify the distribution of cluster sizes we evaluated the cumulative distribution function (CDF) of cluster sizes. We used the same binarized images to perform SIA, which measures the correlation in intensity values *g*(*r*) of each pair of pixels in a given image that are separated by a radial distance *r*^47^, and averages over all pairs with a given *r*. Data shown in Figs. 3,4 and 6 are the average and standard error of *g*(*r*) curves measured across all images at a given time and condition. We fit each *g*(*r*) to an exponential function to quantify a characteristic correlation lengthscale *ξ* associated with the features (e.g., colloids, clusters) in a given image, which we normalize by the colloid diameter to quantify *ξ* in terms of the number of colloids it spans.

We performed sedimentation experiments in borosilicate glass capillaries with 1 mm × 1 mm inner cross-section (Wale Apparatus) that accommodate ~10 μL of sample. Colloidal suspensions were pipetted into the capillaries which were then sealed by adhering glass coverslips to the openings using UV-curable adhesive. The capillaries were mounted vertically, illuminated with a white light LED, and imaged every hour for 36 hours using an iPhone 6s.

### Mathematical Modeling and Simulations:

We simulate the dynamics of the experimental system using Brownian Dynamics implemented in C++^48^, as described in SI. Our system consists of 500 colloidal particles of diameter σ = 1 μm, confined to a two-dimensional box with edge length 50 μm and periodic boundary conditions. The colloids occupy a static area fraction of 16%, set to match experimental conditions by evaluating the fraction of pixels above threshold in binarized experimental images. At the beginning of each simulation, all colloids are separate particles undergoing Brownian diffusion in 2D. When the surfaces of two colloids come within a distance *l* =10 nm of each other (the approximate size of a KaiBC complex^36^), they have a non-zero probability of linking together. We simulate three cases that correspond to our experimental studies: (1) Permanent crosslinking, where, once formed, bonds between colloidal particles are permanent; (2) No crosslinking, where bonds never form between colloidal particles regardless of their proximity; and (3) Oscillatory crosslinking, where bond formation and dissolution follow the oscillatory complexing of KaiB and KaiC. SI Table S1 provides all simulation parameters, their relation to experimental values, and rationale for their choice.

For cases (1) and (3), when a pair of particles are within a center-to-center distance of $r_{0}=\;\sigma \;+l$, they can become crosslinked with a certain probability. This attachment probability at simulation time *t* is $p_{a}=p_{0}\cos^{2}(\pi t/T)$, where *T* = 24 hrs represents the crosslinker oscillation period. The probability amplitude $p_{0}$ is a phenomenological parameter determined from the fluorescence polarization data for pS KaiC (see Fig. 4I). In cases where the particles can unlink, we implement a detachment probability, $p_{a}=p_{0}\cos^{2}(\pi t/T)$, where *n* is the number of bonds (KaiABC crosslinkers) connecting the particle pair under consideration. At the beginning of the simulation, the system has a maximum probability of attachment and a minimum detachment probability to replicate the experimental schedule of the KaiB-KaiC interaction. We run simulations for 69120τ, corresponding to 48 hrs of experimental time (see SI Movie S1, Table S1), and show averages over 5 runs in the results presented in the paper.

## Data Availability

All data will be made freely available upon request.
