# Supplementary material for: Timed material self-assembly controlled by circadian clock proteins
Source: ArXiv. 2024 Mar 21:arXiv:2303.00779v2. Originally published 2023 Mar 1. Preprint. [Version 2] (PMC10002811)
Supplement: Supplement 1 [file NIHPP2303.00779v2-supplement-1.pdf]

Timed material self-assembly controlled by circadian clock proteins

Gregor Leech[1], Lauren Melcher[2†], Michelle Chiu[3†], Maya Nugent[1], Lily Burton[4], Janet Kang[5], Soo Ji Kim[4], Sourav Roy[6], Leila Farhadi[6], Jennifer L. Ross[6], Moumita Das[2,7], Michael J. Rust[5], Rae M. Robertson-Anderson[1]*

[1]Department of Physics and Biophysics, University of San Diego, San Diego, California 92110, United States

[2]School of Mathematical Sciences , Rochester Institute of Technology, Rochester, New York 14623, United States

[3]Graduate Program in Biophysical Sciences, University of Chicago, Chicago, Illinois 60637, United States

[4]Department of Biochemistry and Molecular Biophysics, University of Chicago, Chicago, Illinois 60637, United States

[5]Department of Molecular Genetics and Cell Biology and Department of Physics, University of Chicago, Chicago, Illinois 60637, United States

[6]Department of Physics, Syracuse University, Syracuse, New York 13244, United States

[7]School of Physics and Astronomy, Rochester Institute of Technology, Rochester, New York 14623, United States

[†]These authors contributed equally to this work.

Supplementary Information

Section S1. Supplementary Methods

Figure S1. Biotinylation of KaiB preserves phosphorylation-dependent binding to KaiC.

Figure S2. Confocal microscopy of colloidal suspensions incubated with pT KaiC + 55% b-KaiB (left), pS KaiC + 55% b-KaiB (right), and pS KaiC + 0% b-KaiB (middle).

Figure S3. Complementary image analysis methods quantify selective colloid clustering with pS KaiC.

Figure S4. Time-dependence of KaiC-mediated cluster formation quantified via cluster size analysis.

Figure S5. Clock-mediated crosslinking of 2 µm and 6 µm colloids display the same self-assembly kinetics as 1 µm colloids in the presence of biotinylated Kai proteins.

Figure S6. Brightfield microscopy of suspensions of passivated colloids incubated with pT KaiC (left), pS KaiC (middle), or WT KaiC (right) with 55% b-KaiB.

Figure S7. Streptavidin-coated colloids in the presence of biotinylated PEG or BSA (no Kai proteins), or in the presence of biotin-KaiB and WT KaiB but no KaiC, show no apparent crosslinking or clustering.

Figure S8. The time constant for colloidal crosslinking is robust to the concentration and mirrors the time constant of KaiBC complex formation.

Figure S9. Both timing and extent of salt-mediated clustering of colloids depend on salt concentration.

Movie S1. Animations of simulations of colloids crosslinking under the action of (A) no crosslinkers (N), (B) permanent crosslinkers (P), and in cases in which each connection between two colloids is formed by (C) 1, (D) 2 or (E) 3 oscillatory crosslinkers.

Table S1. Table of parameters used in modeling and simulations.

Supplementary References

Section S1. Supplementary Methods

Protein preparation and characterization

Protein expression and purification: KaiA, KaiB, KaiC, pT KaiC (KaiC-AE; S431A, T432E), and pS KaiC (KaiC-EA; S431E, T432A) were recombinantly expressed and purified as previously described [1,2]. For experiments other than the initial characterization by fluorescence polarization in Fig 2C, WT KaiC carried an N-terminal FLAG epitope and was expressed using a SUMO tag following[3]. Purified proteins were buffer-exchanged into Kai buffer containing: 10% glycerol, 150 mM NaCl, 20 mM Tris-HCl (pH 8.0), 5 mM $MgCl_2$, 0.5 mM EDTA (pH 8.0), 1 mM ATP (pH 8.0). Protein concentration was measured by Bradford Assay (Bio-Rad) using bovine serum albumin (BSA) as a standard.

Biotinylation of KaiB: KaiB stock (70 µM) was buffer-exchanged into labeling buffer (pH 8.0), containing 20 mM HEPES and 150 mM NaCl, using Zeba Spin Desalting Columns with 7KD MWCO (ThermoFisher). KaiB was functionalized with biotin using EZ-Link-Sulfo-NHS-LC-Biotin (ThermoFisher). The biotin reagent was added in 50× molar excess to KaiB, and the reaction was incubated at room temperature for 30 minutes. Unincorporated biotin was then removed using Zeba spin columns equilibrated in Kai buffer.

Pull-down assay of KaiBC complexes using b-KaiB as bait: Reactions with 6.5 µM KaiC (wild-type, pT mutant or pS mutant) were mixed with 5.5 µM KaiB (55% b-KaiB, 45% unlabeled KaiB) in Kai buffer and incubated for 8 hrs at 30°C to allow protein complex formation. The negative control without b-KaiB (6.0 µM unlabeled KaiB) was incubated for 20 hours. b-KaiB and its binding partners were removed from solution by incubation with streptavidin-coated magnetic beads (Cytiva). To prepare the bead slurry, 20 µL of the resin stock was washed twice with 100 µL of Kai buffer. The resulting pellet was resuspended with with 5 µL of the Kai protein reaction. The reaction was incubated for 30 minutes at 22°C with shaking at 950 rpm, after which the beads were magnetically pelleted. The resulting supernatant was transferred into a new tube and analyzed by SDS-PAGE using 4-20% Criterion TGX gels (Bio-Rad) and stained using SYPRO Ruby (Invitrogen).

Fluorescence polarization: To characterize the tolerance of the standard oscillator reaction to b-KaiB, clock reactions were prepared by mixing together 1.5 µM KaiA, 3.5 µM KaiC, different ratios of KaiB and b-KaiB, and 0.2 µM FITC-labeled $KaiB^{K25C}$ in Kai buffer, similar to the procedure used previously[4]. For each reaction, the total concentration of KaiB and b-KaiB was 3.5 µM. 30 µL of the reaction mixture was added to a black 384-well plate, which was sealed with low-autofluorescence polyolefin film (USA Scientific). The plate was loaded into a plate reader to measure fluorescence polarization with 485 nm excitation (20 nm bandpass) and 535 nm emission (25 nm bandpass) using a 510-nm dichroic filter. Measurements were taken every 15 minutes. Wells containing only Kai storage buffer were used as blanks. We performed the same fluorescence polarization assay to characterize protein function under colloid-linking conditions. For these experiments, we mixed together 5.5 µM KaiB (55% b-KaiB, 45% unlabeled KaiB), 6.5 µM KaiC (wild-type, pT, or pS), 2.2 µM KaiA, and 0.4 µM FITC-labeled $KaiB^{K25C}$ in Kai buffer.

Characterization of phosphorylation state of KaiC in the colloidal system: KaiC phosphorylation in each sample was resolved by SDS-PAGE analysis using 10% Criterion Tris-HCl gels (Bio-Rad) run for 3 hrs at 125 V. The gels were stained with SYPRO Ruby (Invitrogen) and imaged using ChemiDoc (Bio-Rad). The ratio of phosphorylated KaiC to unphosphorylated KaiC was quantified by gel densitometry.

Clock-colloid experiments

Colloidal suspensions: We used streptavidin-coated Fluoresbrite YG 1.0-µm diameter polystyrene microspheres (Polysciences) as the colloids in all experiments. The colloids were stored suspended in water at 1.4% solids at 4°C. Immediately prior to each experiment, the colloids were washed and resuspended to 3.7% solids in Kai reaction buffer as follows. 58 µL of the stock suspension was centrifuged in a low-

retention microcentrifuge tube at $11500\times g$ for 5 mins to pellet the colloids. The supernatant was immediately removed and replaced with 80 µL of Kai buffer, followed by vortexing to resuspend the colloids. This washing process is repeated two more times. Following the third spin down, the colloids are resuspended at 3.7% solids in Kai buffer. The suspension was mixed by vortexing and then further homogenized by sonication for 45 mins at 4˚C.

To prepare Kai-colloid suspensions, we add 3.6 µM b-KaiB to the sonicated colloidal suspension, vortex for ~3 secs, and incubate at room temperature for 5 mins to allow b-KaiB to coat the colloids. Next, we mix in 2.9 µM unlabeled KaiB and 2.2 µM KaiA (if using) to create a master mix that we then divided into three tubes to which we add 6.5 µM of either wild-type KaiC, pS KaiC or pT KaiC. $t = 0$ is defined as the time of KaiC addition. The final colloid concentration is 1.26% solids (~4.0×10^{-5} µM) in all cases.

Microscopy samples: Kai-colloid suspensions are flowed via capillary action into passivated sample chambers which are prepared as follows. Glass microscope slides and No. 1 coverslips are cleaned with methanol, rinsed with deionized water, and air dried. Three 22 × 3 mm flow channels are formed by fusing the slide and slip together with heated ~120-µm thick parafilm spacers to accommodate 8 µL of sample in each channel. To prevent non-specific binding of colloids and proteins to the chamber walls, we passivate the surfaces using bovine serum albumin (BSA)[5] as follows. We fill sample chambers with 10 mg/ml BSA in Kai buffer, incubate in a hydrated chamber at room temperature for 30 minutes, then flush the BSA solution out with fresh Kai buffer. Sample chambers are then filled with the colloidal suspension, sealed with UV-curable adhesive, and placed on a 360˚ rotator at 30 ˚C. For each experiment two replicates of each case are prepared and imaged immediately after one another.

Microscopy Experiments: Colloidal suspensions were imaged using an Olympus IX73 epifluorescence microscope with a 40× 0.6 NA objective, 480/535-nm excitation/emission filters, and a Hamamatsu ORCA-Flash 2.8 CMOS camera. 1920×1440 pixel images of suspensions are captured 2-4 µm above the coverslip, in 6 equidistant regions around the horizontal center of sample chambers. Single images were captured sequentially for each sample in the three-lane chamber within a span of five minutes, for a total of 18 images in a 15 min period. This process was then immediately repeated for the replicate chamber. This set of 18 images were captured every ~3 hrs over a 28 hr period. All data shown consists of 2-3 replicate experiments with vertical error bars representing standard error. Horizontal error bars indicate the variation in image acquisition times across replicates binned together into one data point (~15 mins for most cases).

To construct confocal z-stacks shown in Figs 3 and 6, we imaged samples using a Nikon A1R scanning confocal microscope with a 60× 1.4 NA oil-immersion objective. Colloids are imaged using a 488 nm laser with 488/595 nm excitation/emission filters. Stacks of 51 square images, with planar edge lengths of 512 pixels (210 µm), are constructed using a 0.2 µm z-step size, starting from the coverslip ($z = 0$) and going up to $z = 10$ µm.

Image post-processing and analysis: We analyzed images using three different approaches (see Fig 3). All image post-processing and analyses were performed using custom-written Python codes[6–8]. We first evaluated the distribution of pixel intensities across all images for a given time-point and condition, which we normalize to probability density distributions. To account for variations in image brightness across different images we subtract from each pixel value the [mean – minimum] pixel value. We construct histograms using 5000 bins.

We also performed spatial image autocorrelation (SIA) analysis in Fourier space and directly measured 2D cluster sizes in real space. For both analyses, we first binarized images by using local thresholding algorithms with a local block of 1051×1051 pixels[9,10], such that all pixel values are converted to 1 or 0. To measure cluster sizes, we identify each connected set of pixels above threshold as a cluster and count the number of pixels in each such region. By dividing the number of pixels within each 2D area by the maximum cross-sectional area of a colloid, we estimate the size of each cluster in terms of the minimum number of colloids it comprises. To quantify the distribution of cluster sizes we evaluate the cumulative distribution function (CDF) of cluster sizes across all images for a given time-point and condition.

We used the same binarized images to perform SIA, which measures the correlation in intensity values $g(r)$ of each pair of pixels in a given image that are separated by a radial distance r [11], and averages over all pairs with a given r. In practice, we generate $g(r)$ for each image by taking the fast Fourier transform of the image $F\big(I(r)\big)$, multiplying by its complex conjugate, applying an inverse Fourier transform F^{-1} and normalizing by the squared intensity $[I(r)]^2$: $g(r) = [F^{-1}\left|F\big(I(r)\big)\right|^2]/[I(r)]^2$. We generate a separate $g(r)$ curve for each image, and the data shown in Figs 3,4 and 6 are the average and standard error of $g(r)$ across all images at a given time and condition. To quantify a characteristic lengthscale associated with the features (e.g., colloids, clusters) in a given image, we fit the corresponding $g(r)$ to an exponential function, $g(r) = (1 - A)e^{-r/\xi} + A$, where A is a constant that accounts for non-zero asymptotes and ξ is the correlation length that estimates the average size of clusters in an image. To generate the experimental data shown in Figs 4-6, we compute ξ for each image, average over all images for a given condition and time-point, and normalize by the colloid diameter to quantify ξ in terms of the number of colloids it spans.

Sedimentation Experiments: Sedimentation experiments were carried out in borosilicate glass capillaries with 1 mm × 1 mm inner cross-section, 0.2 mm wall thickness and 11 mm length (Wale Apparatus, #8100-050), accommodating ~10 µL of aqueous sample. Capillaries are secured to a 10 mm × 25 mm microscope slide with UV-curable adhesive, oriented such that each end extends ~0.5 mm beyond the slide. The overhanging ends are then sanded so they are flush with the slide. The colloidal suspensions, prepared as described above, are pipetted into the capillaries via capillary action. To seal the capillaries, glass coverslips (7 mm × 22 mm) are adhered to the bottom and top openings using UV-curable adhesive. A second coat of UV-curable adhesiveis added to the junctions between the chambers and coverslip to prevent sample evaporation and leakage.

The samples are mounted vertically against a white background in an enclosed space protected from light. To acquire time-lapse images of the colloidal suspensions undergoing during sedimentation, the mounted capillaries are illuminated with a white light LED and 1024 x 1024 square-pixel images are captured every hour for 36 hours using an iPhone 6s. The capillaries and lighting remain unperturbed for the entire 36-hr acquisition.

Mathematical Modeling and Simulations

Simulation Setup: We simulate the dynamics of the experimental system using Brownian Dynamics implemented in C++[12,13]. Our system consists of 500 colloidal particles of diameter $\sigma = 1$ µm, confined to a two-dimensional box with edge length 50 µm and periodic boundary conditions. The colloids occupy a static area fraction of 16%, set to match the bead density in the imaging plane estimated from the binarized experimental images at $t = 0$. At the beginning of each simulation, all colloids are separate particles undergoing Brownian diffusion in 2D. When the surfaces of two colloids come within a distance l =10 nm of each other[14], they have a non-zero probability of linking together. If a pair of particles gets too close, they experience hard sphere repulsion. We simulate three cases that correspond to our experimental studies: (1) Permanent crosslinking, where, once formed, bonds between colloidal particles are permanent; (2) No crosslinking, where bonds never form between colloidal particles regardless of their proximity; and (3) Oscillatory crosslinking, where bond formation and dissolution follow the oscillatory activation and deactivation of the KaiBC complex. SI Table S1 provides all simulation parameters, their relation to experimental values, and rational for their choice.

Crosslinking Kinetics: For the cases (1) and (3), when a pair of particles are within a center-to-center distance of r_0, they can become crosslinked with a certain probability. This probability of attachment at simulation time t is $p_a = p_0 \cos^2(\pi t/T)$, where T represents the crosslinker oscillation period, and is set to 24 hours commensurate with the ~24-hr rhythm of the Kai clock. The probability amplitude p_0 is a phenomenological parameter, and we determine its value from the fluorescence polarization data for pS KaiC (see Fig 2). We fit an exponential function $y(t) = y_{ss} + (y_0 - y_{ss})e^{-t/\tau_F}$ to this data, where y_0 and

y_{ss} denote the fluorescence polarization value at the onset of the experiment and at time when the system has reached a steady state, respectively. We measure $\tau_F \simeq 4 \times 10^4$ s from this fit, which we use to obtain the corresponding probability $p_0 \simeq 1 - e^{-\tau/\tau_F} \approx \tau/\tau_F$. Here, $\tau = 2.4$ s is the timescale used to non-dimensionalize simulations and is set to the time for a colloid to diffuse across its own diameter in water. In cases where the particles can rhythmically unlink, we implement a detachment probability, $p_d = p_0^n \sin^2(\pi t/T)$, where n is the number of bonds (KaiABC crosslinkers) connecting the particle pair under consideration. At the beginning of the simulation, the system has a maximum probability of attachment and a minimum detachment probability to simulate the KaiABC reaction beginning in a phase corresponding to KaiB-KaiC complex formation. We vary n as a proxy for varying Kai concentration.

Dynamics of colloidal particles: The movement of the ith colloidal particle follows the overdamped Langevin equation, $\frac{d\boldsymbol{r}_i}{dt} = \frac{D}{k_B T}\sum_j\left(\boldsymbol{F}_{c,ij} + \boldsymbol{F}_{LJ,ij}\right) + \sqrt{2D}\boldsymbol{\eta}_{\boldsymbol{i}}$, where k_B is the Boltzmann constant, temperature T is set to room temperature, D is the colloid diffusion coefficient, and η represents Gaussian noise with zero mean and unit variance. The interparticle interaction force consists of two contributions. The first contribution comes from an elastic spring force $\boldsymbol{F}_{c,ij}$ due to stretching or compressing the crosslinker that connects particles i and j, defined as $\boldsymbol{F}_{c,ij} = -K\left(r_{ij} - r_0\right)\hat{r}$, where K is the spring constant, r_{ij} is the distance between the centers of particles i and j, and r_0 is the sum of particle diameter σ and crosslinker rest length. The second contribution comes from the hard-sphere interaction between particles, modeled by the repulsive part of the 12-6 Lennard-Jones potential, $V_{LJ} = 4\epsilon\left[\left(\frac{\sigma}{r_{ij}}\right)^{12} - \left(\frac{\sigma}{r_{ij}}\right)^{6}\right]$, where ϵ is the interaction strength. We non-dimensionalize equations by scaling distances by the particle diameter σ, times by the diffusion timescale $\tau = \sigma^2/D$, and energies by $k_B T$. We run simulations for 69120τ, corresponding to 48 hrs of experimental time (see Table S1), and show averages over 5 runs in the results presented in the paper.

Spatial image autocorrelation (SIA): We performed the same SIA algorithms used to analyze experimental images on system configuration images from simulations generated using MATLAB at time points corresponding to the experimental time points of 1, 7, 14, 21 and 28 hrs. Prior to SIA analysis, image files are converted to binary images with colloids being filled white circles and the empty space being black, similar to experimental binarized images. To determine the average $g(r)$ and ξ for each time point, we analyzed images for 5 consecutive time points centered on the time of interest, consistent with experiments. $g(r)$ and ξ values shown in Figs 5 and 6 are averages of the values determined from the 5 different images with error bars representing standard error.

Simulation animations: Animations of the simulations for all 5 crosslinking cases considered in the study, provided in SI, were rendered using an OpenGL (Open Graphics Library) script written in MATLAB. The packages used to run them were GCC (C/C++) and ffmpeg, and the inputs were the colloid center positions and colloid IDs that indicated which colloids were crosslinked.

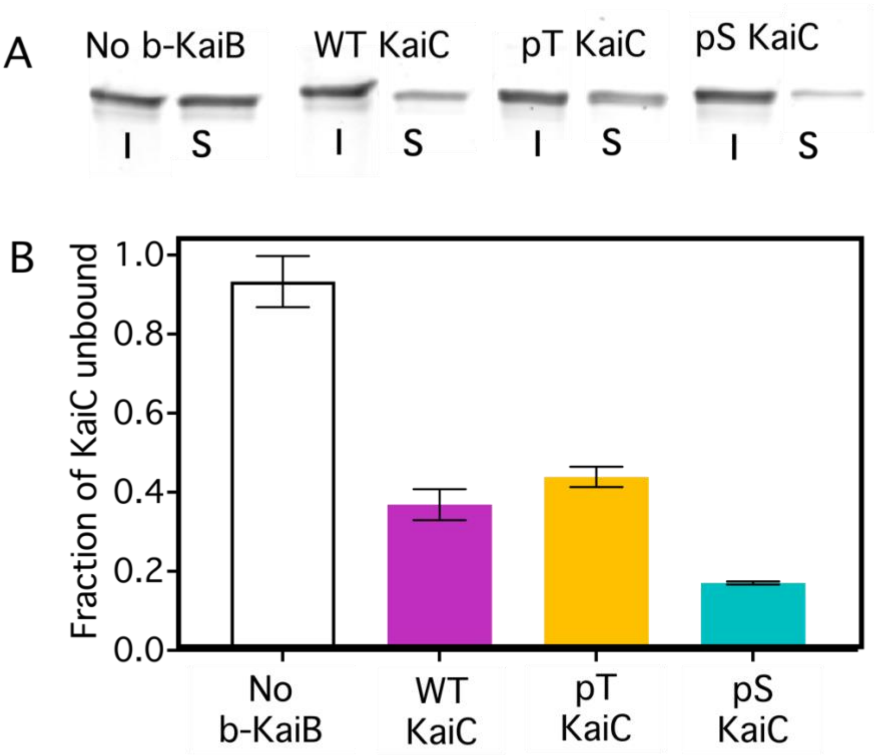

Figure S1. Biotinylation of KaiB preserves phosphorylation-dependent binding to KaiC. Streptavidin pull-down analysis of the KaiB-KaiC interaction with and without biotinylated KaiB (b-KaiB). Data from left to right are for: wild-type KaiC with 100% wild-type KaiB (No b-KaiB, white), wild-type KaiC with 55% b-KaiB and 45% KaiB (WT KaiC, magenta), non-binding pT KaiC with 55% b-KaiB and 45% KaiB (pT KaiC, yellow), and constitutively-binding pS KaiC with 55% b-KaiB and 45% KaiB (pS KaiC, cyan). **(A)** SDS-PAGE gel of KaiC remaining in the supernatant (S), and thus not in complex with b-KaiB, following pull-down using magnetic streptavidin-coated beads. Each pair of bands corresponds to KaiC in the input reaction after 8-hr incubation (I) and supernatant following pull-down (S). **(B)** Ratios of the supernatant and input determined by gel densitometry for each pair shown in (A), with 0 indicating that all KaiC is bound to b-KaiC while 1 indicates no KaiC is bound.

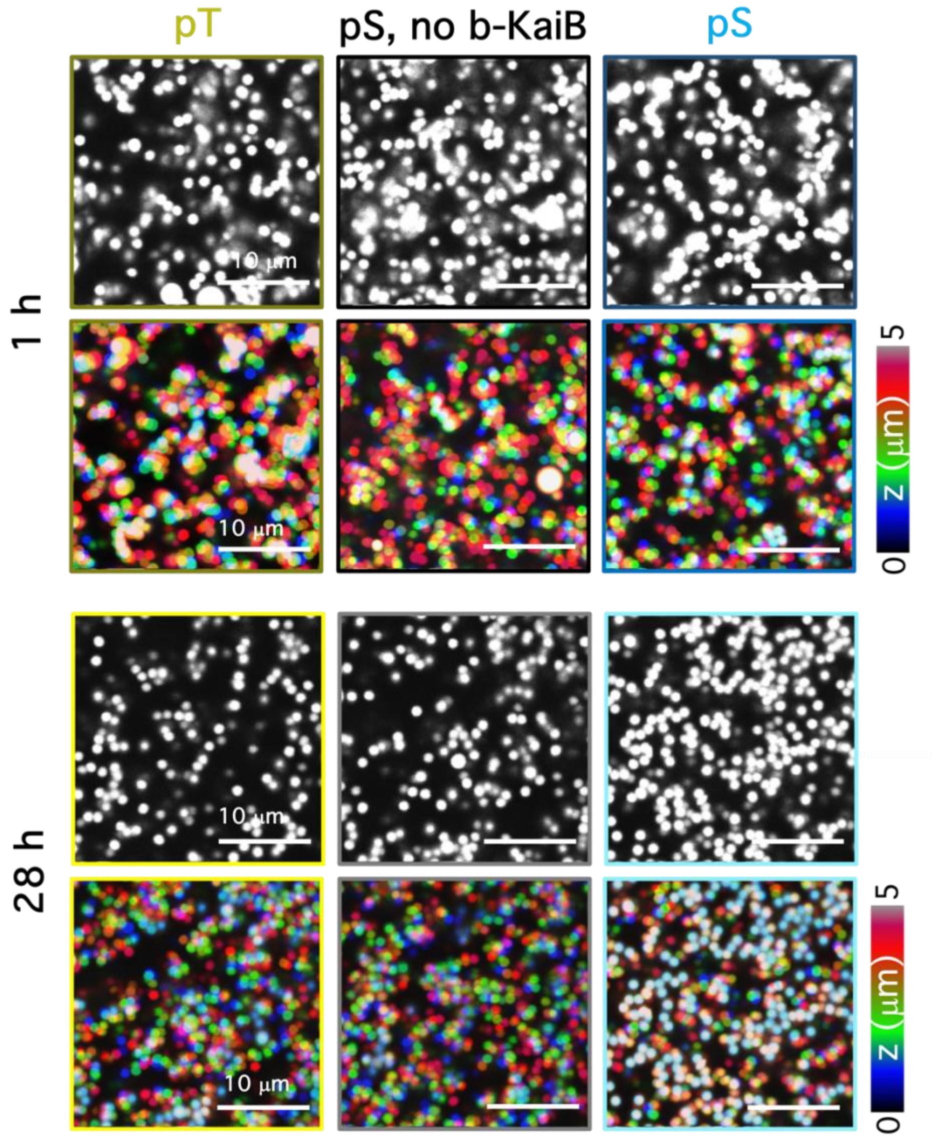

Figure S2. Confocal microscopy of colloidal suspensions incubated with pT KaiC + 55% b-KaiB (left), pS KaiC + 55% b-KaiB (right), and pS KaiC + 0% b-KaiB (middle). Confocal images (rows 1,3) and *z*-projections (rows 2,4) of colloidal suspensions incubated with 6.5 µM mutant KaiC (pT or pS) and 6.5 µM KaiB for 2 hrs (rows 1,2) and 28 hrs (rows 3,4). The left and right columns are with pT KaiC and pS KaiC, respectively, and 55% of the KaiB is b-KaiB (as in the experiments in the main text). The middle column includes the constitutively binding pS KaiC mutant but none of the KaiB is biotinylated. The left and middle columns are nearly indistinguishable from one another and show minimal clustering, indicating that the crosslinking is specifically mediated by b-KaiB-KaiC interactions. The right column shows more clustering of colloids and hindered motion, seen as more white regions in the z-projections. Data acquisition and imaging parameters are described in Methods and SI Methods.

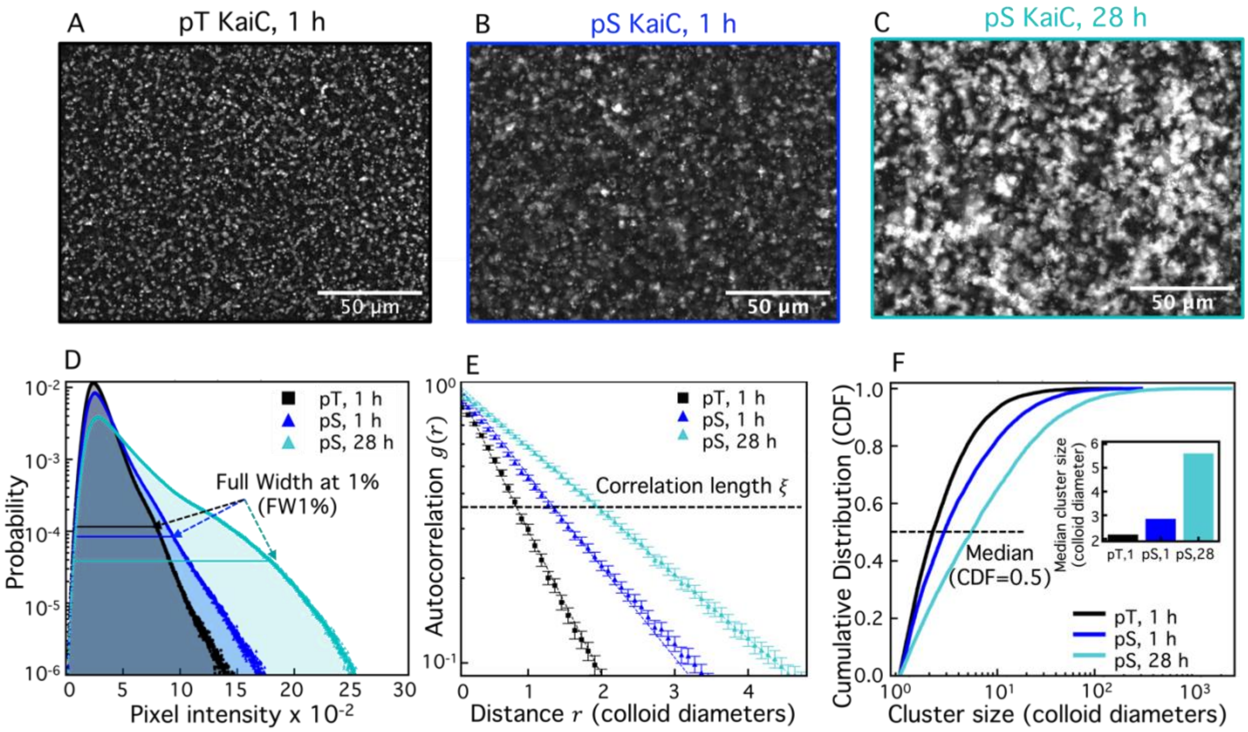

Figure S3. Complementary image analysis methods quantify selective colloid clustering with pS KaiC. **(A-C)** Fluorescence microscopy images of 1-µm colloids taken at 1 h (A,B) and 28 h (C) after mixing with KaiC mutants that are frozen in non-binding (A, pT) or binding (pS, B,C) states. **(D-F)** Quantification of colloidal clustering in epifluorescence images by evaluating **(D)** the pixel intensity distributions of images, **(E)** spatial image autocorrelation (SIA) function $g(r)$, i.e., the correlation between two pixels separated by a radial distance r, and **(F)** the normalized cumulative distribution (CDF) of cluster sizes, where a cluster is defined as a connected set of above-threshold pixels. To compare these complementary analyses, we compute **(D)** the full width of each intensity distribution at 1% of the corresponding mode, denoted by the horizontal lines; **(E)** the characteristic correlation length ξ, determined by fitting each $g(r)$ curve to an exponential function (color-coded dashed lines), and denoted by the corresponding intersection of the horizontal dashed line at $g = e^{-1}$; **(F)** the cluster size at CDF = 0.5, i.e., the median cluster size, as denoted by the intersection of the horizontal dashed line with each CDF. The data shown in D-F are from images collected at 1 h for pS (black) and pT (blue) and 28 hrs for pS (cyan). Further details regarding the analyses depicted in D-F are described in Methods and SI Methods.

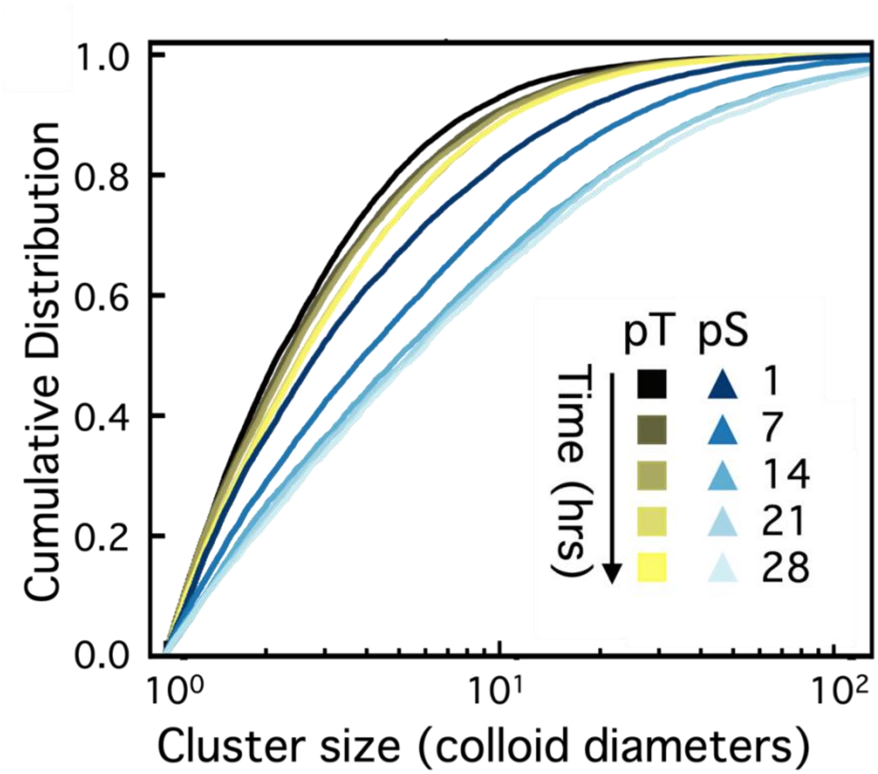

Figure S4. Time-dependence of KaiC-mediated cluster formation quantified via cluster size analysis. (A) CDFs of cluster sizes for 5 different times between 1 hr and 28 hr for pT (yellow) and pS (cyan) with color shade indicating time according to the legend. CDFs are used to determine median cluster sizes plotted in Fig 3, as depicted in Fig S3F.

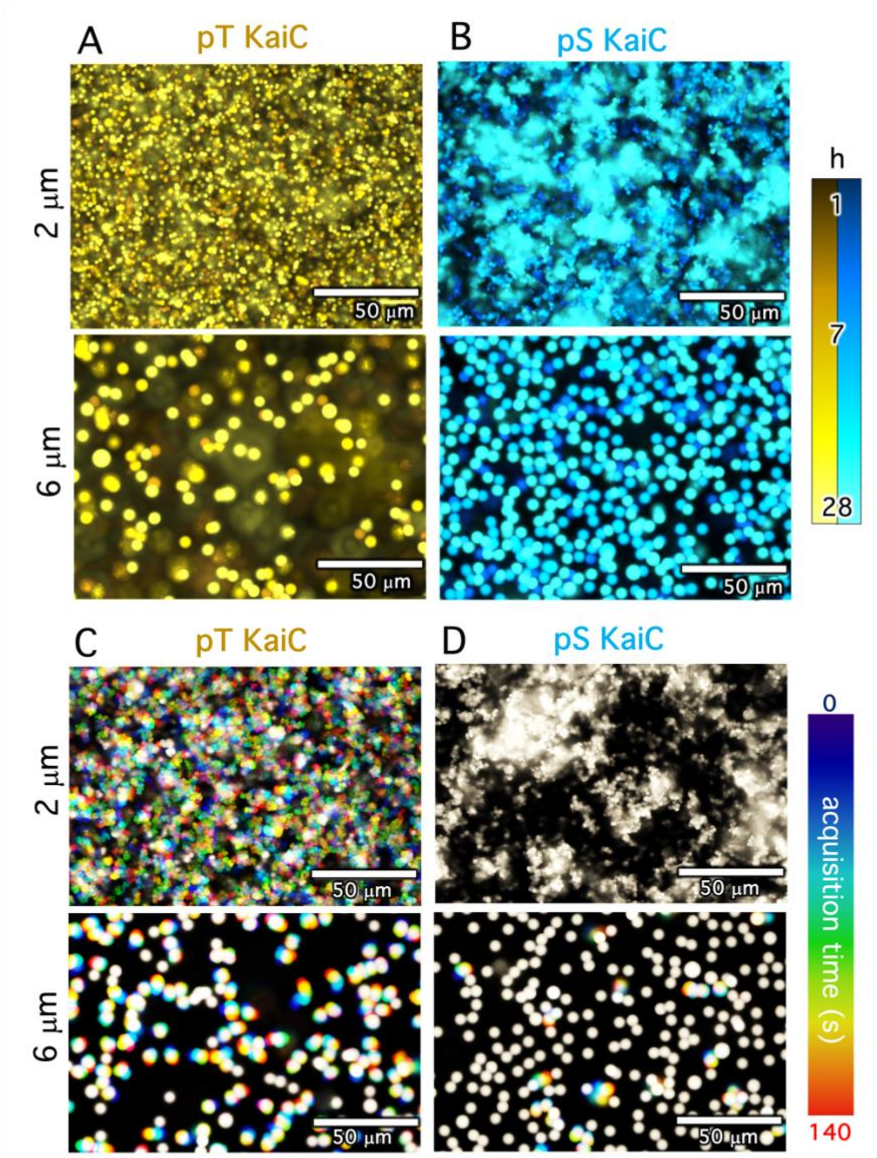

Figure S5. Clock-mediated crosslinking of 2 µm and 6 µm colloids display the same self-assembly kinetics as 1 µm colloids in the presence of biotinylated Kai proteins. (**A,B**) Colorized temporal overlays of fluorescence microscopy images taken 1 h, 7h, and 28 h (dark to light) after mixing 2 µm (top) and 6 µm (bottom) colloids with pT KaiC (A, yellow) and pS KaiC (B, cyan) show cluster growth over time with pS that is absent for pT. (C, D) Temporal color-coded collapses of 140-s videos taken at 28 h for 2 µm (top) and 6 µm (bottom) colloids in the presence of pT (C) or pS (D). White indicates lack of motion due to the exact overlay of all colors corresponding to different frames. Colloids in the presence of pT maintain mobility, seen as more color in the images, while pS induces aggregation and crosslinking that stalls colloid motion. The concentrations of colloids, proteins and reagents, as well as imaging parameters, are identical to those for the 1 µm colloid experiments shown in Figs 2-4. As shown, timed aggregation, dependent on the phosphorylation state of KaiC is preserved for different sizes of colloids.

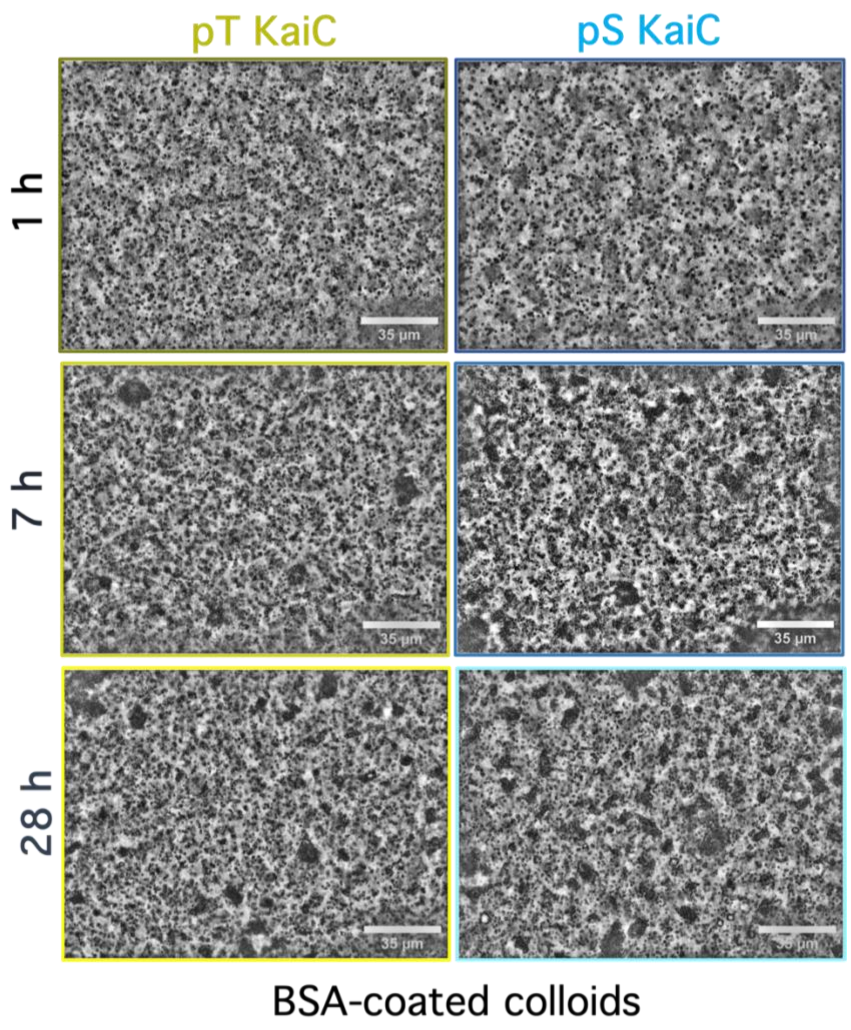

Figure S6. Brightfield microscopy of suspensions of passivated colloids incubated with pT KaiC (left) or pS KaiC (right) with 55% b-KaiB. Brightfield microscopy images of colloids of the same size and material (1-µm, polystyrene, Polysciences, Inc) as streptavidin-coated colloids used in the Kai-colloid crosslinking experiments but coated with BSA rather than streptavidin. Images show minimal clustering and aggregation for both pT and pS over the course of 28 hrs. The modest clumping observed at later times is not specific to the type of KaiC, demonstrating that the Kai-specific timed self-assembly of colloids that we observe in experiments is due to specific interactions between KaiC and colloid-bound KaiB proteins. KaiC concentration in all experiments is 6.67 µM, the highest concentration used in experiments in the main text. Imaging parameters are the same as in the main text but brightfield instead of fluorescence is used because the colloids are not fluorescently-labeled.

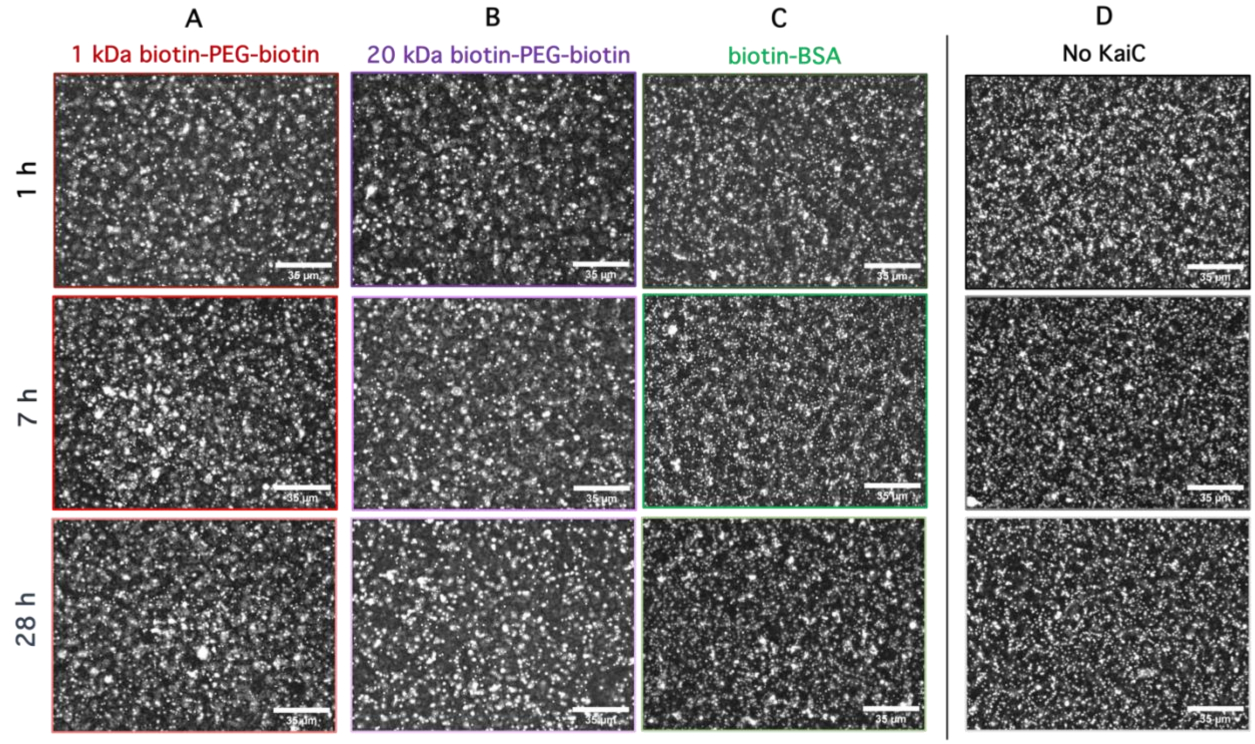

Figure S7. Streptavidin-coated colloids in the presence of biotinylated PEG or BSA (no Kai proteins), or in the presence of biotin-KaiB and WT KaiB but no KaiC, show no apparent crosslinking or clustering. Suspensions of streptavidin-coated colloids, with identical conditions to those shown in the main text, but with (**A-C**) Kai proteins replaced with different biotinylated constructs that could, in principle, crosslink streptavidin-coated colloids. To demonstrate that the timed crosslinking we observe is specific to the Kai clock proteins, we replaced Kai proteins with different constructs that have multiple biotins to allow for potential crosslinking of streptavidin-coated colloids: (**A**) 1 kDa biotin-PEG-biotin (PG2-BN-20k, Nanocs Inc), (**B**) 20 kDa biotin-PEG-biotin (PG2-BN-1k, Nanocs Inc), and (**C**) biotinylated BSA, with 8-16 biotins per BSA (A8549, Sigma-Aldrich). Both PEGs have biotin labels on either end and only differ in the lengths of the PEG linker (1 vs 20 kDa). BSA has 8-16 randomly distributed biotins per BSA molecule. In the images shown, the molarity of the constructs was matched to the highest KaiC molarity used in Kai-crosslinking experiments. As shown, no discernible crosslinking or clustering is observed over the course of 28 hrs for all biotinylated linkers. (D) Experiments performed identically to those in the main text but in the absence of KaiC (only biotin-KaiB and WT KaiB) show no discernible crosslinking, demonstrating that the complexation of KaiC with KaiB is essential to colloidal self-assembly. Top and bottom row images are the same as in Fig 2F,G.

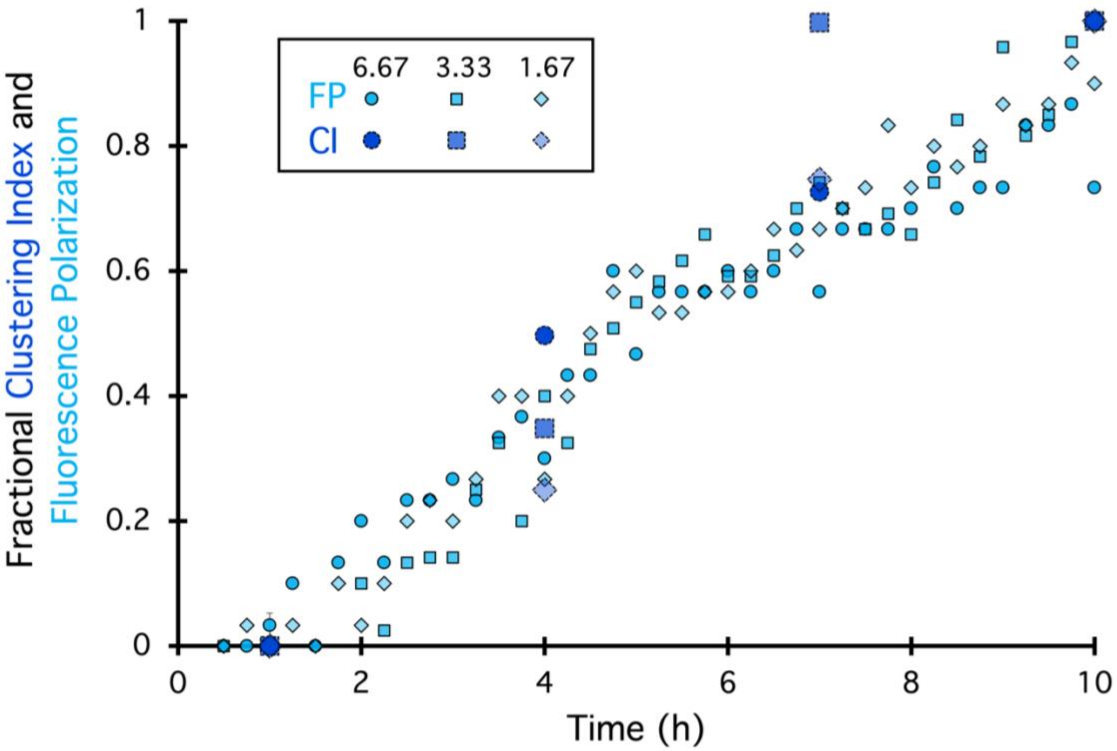

Figure S8. The time constant for colloidal crosslinking is robust to the concentration and mirrors the time constant of KaiBC complex formation. Time-dependent fractional clustering index (CI, blue, dashed borders), determined from the SIA correlation length ξ as described in the main text, for 1 µm colloids in the presence of varying concentrations of pS KaiC: 6.67 µM (circles), 3.33 µM (squares) and 1.67 µM (diamonds). Fluorescence polarization (FP, cyan, solid borders), an indicator of KaiBC complex formation, measured for the same Kai concentrations as the colloid experiments, is also plotted using smaller symbols. These data demonstrate that the rate of the slow self-assembly of colloids in the presence of pT KaiC is independent of Kai concentration, as is the rate of KaiBC complex formation. Moreover, the time-courses of CI and FP data are highly similar, a strong indicator that the time constant for colloidal self-assembly is controlled by the rate of KaiBC assembly.

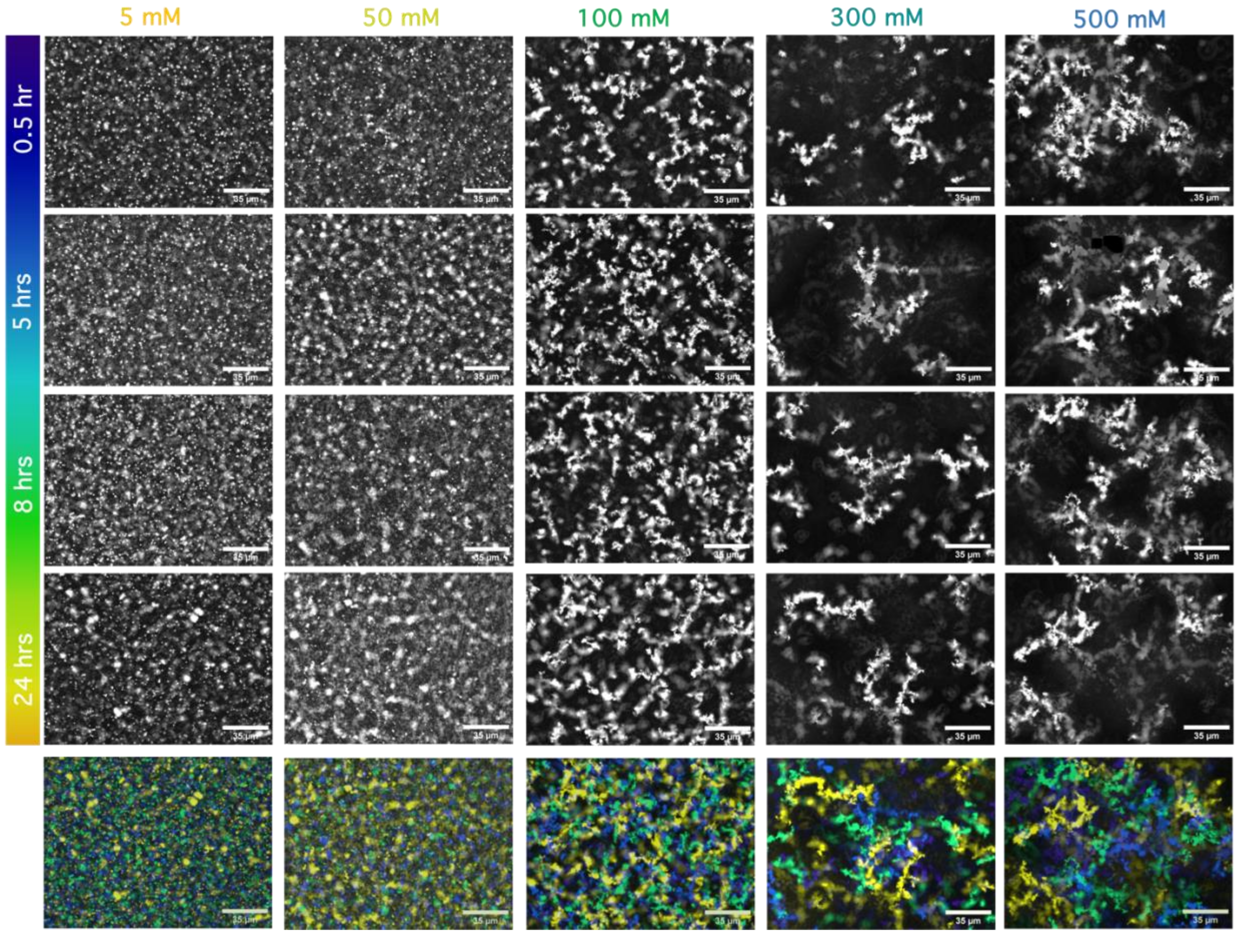

Figure S9. Both timing and extent of salt-mediated clustering of colloids depend on salt concentration. Streptavidin-coated colloids in the same buffer conditions as Kai-crosslinking experiments but in the absence of Kai proteins and with varying added concentrations of $MgCl_2$, listed from left to right. Images were collected 0.5 hr (top), 5 hrs (row 2), 8 hrs (row 3) and 24 hrs (row 4) after adding $MgCl_2$. Bottom row images are overlays of images from all 4 time points, color-coded according to time based on the color scale to the left. Increasing [$MgCl_2$] causes increasing degrees of aggregation and clustering. The timing of the onset and saturation of aggregation also varies with [$MgCl_2$]. At 5 mM, only small-scale clustering is observed starting at ~8 hrs and continuing to increase without saturating up to 24 hrs. At 50 mM, more clustering is observed and the onset is earlier (~5 hrs) than with 5 mM, but no saturation is apparent. For [$MgCl_2$]≥100 mM, the onset of clustering is immediate and appears to saturate at ~5 hrs (100 mM) or sooner (300 mM, 500 mM). The timing of clustering appears to be directly linked to the salt concentration, unlike the timing of clock-mediated crosslinking.

Movie S1. Animations of simulations of colloids crosslinking under the action of (A) no crosslinkers (N), (B) permanent crosslinkers (P), and in cases in which each connection between two colloids is formed by (C) 1, (D) 2 or (E) 3 oscillatory crosslinkers. Animations show the system dynamics over the course of 48 hrs with 1 μm colloids shown as red circles and bonds shown as purple connecting lines. The timestamp in the bottom left lists time in hours. Images depict a 2D square box of length 50 μm in which the colloids are confined.

(A) No Bond
(B) Permanent Bond
(C) 1 Oscillatory Bond
(D) 2 Oscillatory Bonds
(E) 3 Oscillatory Bonds

Table S1. Table of parameters used in simulations. Key parameters and their values used in the model and simulations. Numerical estimates of all parameters are based on experimental values as described in the Source/Rationale column.

**Simulation Parameters**	**Value**	**Source/Rationale**
2D colloid packing fraction, p_f	16%	Area fraction of pixels above threshold in binarized images of pT colloids
Simulation unit lengthscale, σ	1 µm	Diameter of colloidal particle
Simulation unit timescale, τ	2.5 s	Time for colloid to diffuse across its diameter in water, $\tau = \sigma^2/D$
Crosslinker oscillation period, T	34560τ	~24 hrs, set to match KaiABC period
Brownian dynamics timestep, dt	0.001τ	Ensures numerically stability
Characteristic timescale of KaiB-KaiC interaction, τ_F	4×10^4 s	Obtained by fitting experimental FP data for pS (Fig 4) to an exponential
Probability of colloid crosslinking, p_0	6e-5	$p_0 \simeq 1 - e^{-\tau/\tau_F} \approx \tau/\tau_F$
Equilibrium distance between centers of crosslinked colloids, r_0	1.01σ	Sum of colloid diameter and ~10 nm size of KaiBC complex
Spring constant of crosslinks, K	$9k_BT/\sigma$	Phenomenological estimate

Supplementary References

1. Lin, J., Chew, J., Chockanathan, U. & Rust, M. J. Mixtures of opposing phosphorylations within hexamers precisely time feedback in the cyanobacterial circadian clock. *Proceedings of the National Academy of Sciences* **111**, E3937–E3945 (2014).
2. Phong, C., Markson, J. S., Wilhoite, C. M. & Rust, M. J. Robust and tunable circadian rhythms from differentially sensitive catalytic domains. *Proceedings of the National Academy of Sciences* **110**, 1124–1129 (2013).
3. Chavan, A. G. *et al.* Reconstitution of an intact clock reveals mechanisms of circadian timekeeping. *Science* **374**, eabd4453 (2021).
4. Leypunskiy, E. *et al.* The cyanobacterial circadian clock follows midday in vivo and in vitro. *eLife* **6**, e23539 (2017).
5. Ma, G. J., Ferhan, A. R., Jackman, J. A. & Cho, N.-J. Conformational flexibility of fatty acid-free bovine serum albumin proteins enables superior antifouling coatings. *Commun Mater* **1**, 1–11 (2020).
6. Leech, G. gregorleech/best-SIA-image-analysis. (2022).
7. Leech, G. gregorleech/best-cluster-analysis. (2022).
8. McGorty, R. rmcgorty/ImageAutocorrelation. (2021).
9. van der Walt, S. *et al.* scikit-image: image processing in Python. *PeerJ* **2**, e453 (2014).
10. Thresholding — skimage v0.19.2 docs. https://scikit-image.org/docs/stable/auto_examples/applications/plot_thresholding.html.
11. Robertson, C. & George, S. C. Theory and practical recommendations for autocorrelation-based image correlation spectroscopy. *J Biomed Opt* **17**, 080801 (2012).
12. Melcher, L. *et al.* Sustained order–disorder transitions in a model colloidal system driven by rhythmic crosslinking. *Soft Matter* **18**, 2920–2927 (2022).
13. Redner, G. S., Hagan, M. F. & Baskaran, A. Structure and Dynamics of a Phase-Separating Active Colloidal Fluid. *Phys. Rev. Lett.* **110**, 055701 (2013).
14. Snijder, J. *et al.* Structures of the cyanobacterial circadian oscillator frozen in a fully assembled state. *Science* **355**, 1181–1184 (2017).
